# Two genomic regions associated with fiber quality traits in Chinese upland cotton under apparent breeding selection

**DOI:** 10.1038/srep38496

**Published:** 2016-12-07

**Authors:** Junji Su, Libei Li, Chaoyou Pang, Hengling Wei, Caixiang Wang, Meizhen Song, Hantao Wang, Shuqi Zhao, Chi Zhang, Guangzhi Mao, Long Huang, Chengshe Wang, Shuli Fan, Shuxun Yu

**Affiliations:** 1 State Key Laboratory of Cotton Biology, Institute of Cotton Research of CAAS, 455000, Anyang, China; 2 College of Agronomy, Northwest A&F University, 712100 Yangling, China; 3 Bioinformatics Division, Biomarker Technologies Corporation, 101300 Beijing, China

## Abstract

Fiber quality is one of the most important agronomic traits of cotton, and understanding the genetic basis of its target traits will accelerate improvements to cotton fiber quality. In this study, a panel comprising 355 upland cotton accessions was used to perform genome-wide association studies (GWASs) of five fiber quality traits in four environments. A total of 16, 10 and 7 SNPs were associated with fiber length (FL), fiber strength (FS) and fiber uniformity (FU), respectively, based on the mixed linear model (MLM). Most importantly, two major genomic regions (MGR1 and MGR2) on chromosome D_t_7 and four potential candidate genes for FL were identified. Analyzing the geographical distribution of favorable haplotypes (FHs) among these lines revealed that two favorable haplotype frequencies (FHFs) were higher in accessions from low-latitude regions than in accessions from high-latitude regions. However, the genetic diversity of lines from the low-latitude regions was lower than the diversity of lines from the high-latitude regions in China. Furthermore, the FHFs differed among cultivars developed during different breeding periods. These results indicate that FHs have undergone artificial selection during upland cotton breeding in recent decades in China and provide a foundation for the further improvement of fiber quality traits.

Cotton (*Gossypium* spp.) is an important crop that provides natural textile fiber and oilseed for human consumption. The cultivated types of cotton include two diploids, *G. herbaceum* L. (2*n* = 2*x* = 26) and *G. arboreum* L. (2*n* = 2*x* = 26), and two tetraploids, *G. hirsutum* L. (2*n* = 4*x* = 52) and *G. barbadense* L. (2*n* = 4*x* = 52). *G. hirsutum* is the most widely cultivated tetraploid cotton species and accounts for 90% of annual worldwide cotton production. *G. hirsutum* (upland cotton) is thought to have originated by hybridization between a maternal Old World “A” genome taxon resembling *G. herbaceum* or *G. arboreum* and paternal New World “D” genome taxon resembling *G. raimondii*^1^. Consequently, the chromosomes of upland cotton are often numbered in two sets of 13, A1 through A13 and D1 through D13; alternatively, the chromosomes may be numbered as 1 through 26, of which numbers 1 through 13 correspond to A1 through A13 and numbers 14 through 26 correspond to D1 through D13. Upland cotton is characterized by its high yield, wide adaptability, and acceptable fiber quality. With increases in global human consumption levels and spinning machine speeds, the need to improve fiber quality is increasing rapidly. Fiber quality traits have been found to be governed by many quantitative trait loci (QTLs) in upland cotton^2^,^3^, and these traits are negatively correlated with yield^4^ and early maturity traits^5^. However, yield and earliness are also important traits that increase the attractiveness of cotton varieties to growers in China. However, it is extremely challenging to improve fiber quality traits without compromising other important characteristics. To overcome these limitations and further improve the fiber quality of cotton, the major QTL alleles associated with the target traits of upland cotton must be identified.

The identification of QTLs governing complex traits has traditionally been facilitated by a linkage analysis approach using segregating biparental populations. Many QTLs related to fiber quality traits have been tagged using molecular markers in intraspecific segregating populations of upland cotton^6^,^7^,^8^,^9^,^10^. A total of 721 QTLs that control fiber quality traits in tetraploid cotton have been reported and are distributed across all 26 chromosomes^3^. Despite numerous studies that have conducted QTL mapping, the gene(s) underlying fiber quality are poorly understood because the roughly estimated QTL intervals extend over several centimorgans, which is a genetic distance that translates into large genomic regions of dozens of megabases. Fortunately, this intrinsic limitation of the QTL mapping approach can be overcome by association mapping panels, which are composed of unrelated lines that have accumulated a far greater number of crossing-over events over the history of breeding. Association mapping of fiber quality traits with simple sequence repeat (SSR) markers has been employed widely for upland cotton, and some SSR markers associated with fiber quality have been detected in succession^11^,^12^,^13^,^14^. However, these studies were limited by the number of polymorphic SSR markers utilized, and candidate gene(s) underlying fiber quality traits have not been identified by this mapping method. In recent years, the rapid development of genome sequencing technology has allowed genome-wide association studies (GWASs) to overcome the aforementioned limitations of QTL mapping and association analyses, and loci associated with many important target traits in plant species have been identified^15^. In addition, the identification of candidate genes has been achieved using the GWAS approach in several plant species, including *Arabidopsis*^16^, rice^17^ and soybean^18^. Recently, high throughput next-generation sequencing technologies such as genotyping-by-sequencing (GBS)^10^, restriction site associated DNA sequencing (RAD-seq)^19^ and specific-locus amplified fragment sequencing (SLAF-seq)^20^,^21^ have provided an opportunity to obtain the required marker coverage in upland cotton cultivars/accessions.

A high-density single-nucleotide polymorphism (SNP) marker map not only provides a resource for QTL linkage mapping and GWASs in cotton but also facilitates the detection of genetic changes associated with cotton domestication and improvement. Domesticated crops have undergone strong human-mediated selection aimed at developing high-yield, superior-quality and stress-tolerant cultivars that are adapted to diverse environmental conditions and agricultural practices^22^,^23^. To improve genomic selection models, the detection of target loci under selection during crop improvement is critical^24^. Patterns of genetic differentiation based on genome sequence comparisons between populations have recently been applied to detect targets of selection in rice^25^, wheat^23^, maize^26^ and sorghum^27^,^28^,^29^. However, the impact of selection on the patterns of genetic variation underlying fiber quality improvement in cotton remains largely unknown.

Despite being the largest cotton-growing nation, China does not domesticate upland cotton. Most upland cotton cultivars planted in China were derived from several germplasm sources, such as Deltapine (DPL), Stoneville (STV), Foster and King, all of which were introduced from the USA^13^. These cultivars represent the foundation of the Chinese cotton breeding program and have played an important role in the development of Chinese upland cotton cultivars^13^. To meet the demands of spinning speeds, the fiber quality traits of Chinese cultivars have been improved to an extent in upland cotton breeding. This practice raises a number of questions, such as which genetic loci or genomic regions control fiber quality traits; when and where these genetic changes occurred during Chinese upland cotton breeding; and whether these loci or genomic regions were selected artificially. Uncovering the underlying pattern of genetic change and the targets of fiber quality trait selection during cotton breeding over the past several decades would answer these questions. In our study, over 81,000 SNP markers were identified and genotyped using an SLAF-seq approach in a diversity panel consisting of 355 upland cotton accessions. In addition, a GWAS approach was used to identify SNP loci or the major genomic regions associated with fiber quality traits in upland cotton. Furthermore, to detect the associated loci or regions subject to selection during breeding, the favorable haplotype frequency (FHF) and genetic diversity were compared among cultivars of different geographical areas and breeding periods. These results will not only lay the foundation for fiber quality trait improvement through marker-assisted breeding but also help us understand the impact of targeted selection on cotton fiber quality improvement and domestication.

## Results

### Phenotypic characteristics of fiber quality

**Figure 1:**
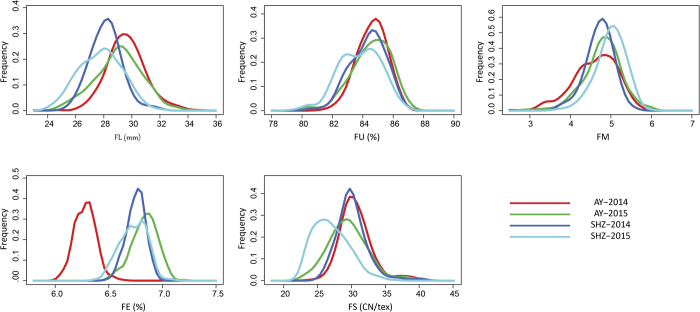
Frequency of the five fiber quality traits of 355 upland cotton accessions. FL: fiber length; FU: fiber uniformity; FM: fiber micronaire; FE: fiber elongation; and FS: fiber strength.

A panel comprising 355 upland cotton accessions was established, and phenotype identification was conducted to study the distribution of five fiber quality traits: fiber length (FL), fiber uniformity (FU), fiber micronaire (FM), fiber elongation (FE) and fiber strength (FS). The diagrams revealed broad variation and a normal distribution without any significant skewness and kurtosis for the five fiber quality traits under four different conditions (Fig. 1). Among the 355 upland cotton lines, the FL ranged from 23.25 to 34.59 mm and had an average value of 28.63 mm and the FU ranged from 79.40% to 87.15% and had an average value of 84.32%. The FU showed continuous variation and ranged from 2.52 to 6.00, with a mean value of 4.77, and the FE ranged from 6.03% to 7.10%, with a mean value of 6.65%. The FS presented a wide range of 22.70–40.65 cN/tex, with a mean value of 29.33 cN/tex. The coefficients of variation (CV) of the FL, FU, FM, FE and FS were 5.01%, 1.44%, 9.07%, 1.60% and 8.83%, respectively (Table S1). Several significant correlations were observed between these five traits. The FL exhibited a highly significant positive correlation with the FS (0.86**), FU (0.76**) and FE (0.74**), whereas several negative correlations were observed between the FM and the other four traits (Table S2).

### Genetic diversity and population structure

The SLAF-seq approach was used to genotype the natural population as described in a previous study^30^, and 691,978 SNPs were identified with call success. A total of 81,675 SNPs were selected for further analyses after excluding the SNPs with more than 10% missing data, a minor allele frequency (MAF) <5%, and an average marker density of 1 SNP per 24.85 kb^30^. To estimate the genetic diversity of natural populations, these SNPs were divided into 26 groups according to chromosome, and the genetic diversity values were calculated for each group. The A subgenome of genetic diversity values ranged from 0.3485 to 0.3897 and had an average value of 0.3656, whereas the D subgenome of genetic diversity values ranged from 0.3465 to 0.4056 and had a mean of 0.3796 (Table S3). The results showed that there is low genetic diversity among Chinese upland cotton.

**Figure 2:**
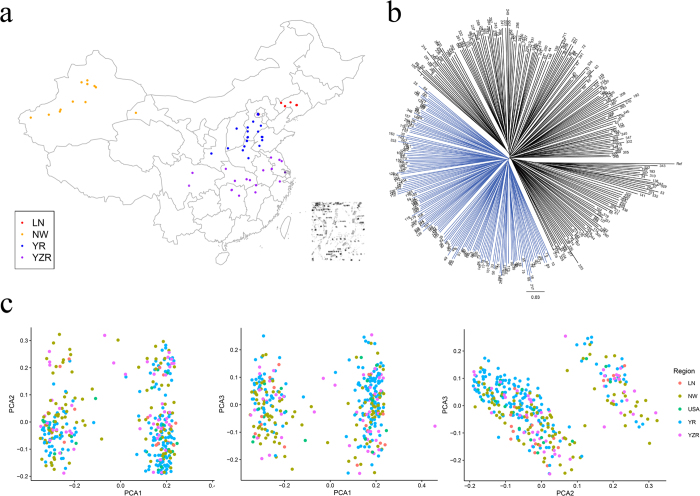
Genetic diversity and population structure of 355 upland cotton genotypes. (**a**) Geographic origin of 331 Chinese upland cotton accessions for which source locations are known; the map of geographic positions of these accessions was generated using the R software package ‘maptools’ (http://r-forge.r-project.org/projects/maptools/), and the source locations are labeled by the color-coded dots. (**b**) Phylogenetic tree constructed by the neighbor-joining method. (**c**) PCA plots of the first three components of population structure, color-coded by geographical origin. YR: 162 accessions from the Yellow River region in China; YZR: 51 accessions from the Yangtze River region in China; NW: 98 accessions from the Northwest Inland region in China; LN: 20 accessions from Liaoning province in China; and USA: 20 accessions from the Texas cotton region in the United States of America.

To represent the genetic, geographic and morphological diversity of Chinese upland cotton, the population included 331 cultivars and new strains gathered from multiple geographic regions across China (Fig. 2a). The pairwise genetic distances among the 355 upland cotton genotypes were determined using SNP markers. A phylogenetic tree based on these genetic distances showed that the genotypes could be classified into two divergent groups (Fig. 2b). Furthermore, a principal component analysis (PCA) was conducted with all selected SNP markers, and two major subpopulations were identified by principal components 1–3 (PC1-3), although certain accession genotypes overlapped (Fig. 2c). PC1 explained 18.09% of the variation in the genotypic data, whereas PC2 and PC3 explained 13.21% and 7.66% of the variation, respectively. The accessions in each group were further classified into several subpopulations, which did not exhibit evident geographic distribution patterns. We also found that the upland cotton accessions were derived from a mixed ancestry, indicating that these lines might have experienced introgression or gene flow during breeding in China.

### Genome-wide association studies (GWASs)

**Figure 3:**
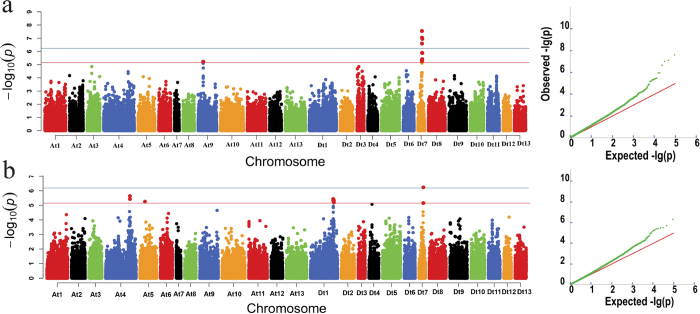
Genome-wide association studies (GWASs) of 355 upland cotton accessions. Manhattan and quantile-quantile plots of GWASs using the mixed linear model (MLM) for fiber length (**a**) and fiber strength (**b**), respectively. The SNP loci of the red lines (−log_10_(*p*) ≥ 5.21) were considered suggestive association markers; The SNP loci of the blue lines (−log_10_(*p*) ≥ 6.21) were considered significant association markers.

GWASs were conducted for five fiber quality traits using the best linear unbiased predictions (BLUPs) of individual performance over four environments in an MLM, which accounts for both population structure and familial relatedness (PCA + K). A total of 16, 10 and 7 SNPs were associated with FL, FS and FU, respectively, whereas no SNPs were associated with FM and FE. For FL, three genomic regions (D_t_7:25767969-25768030, D_t_7:25931988-25999761 and D_t_7:27425475-27437213) on chromosome D_t_7 and a single region (A_t_9:31687000-31778023) on chromosome A_t_9 showed marker-trait associations. Four SNP loci within these associations (rsD_t_7:25931988, rsD_t_7:25932026, rsD_t_7:27437213 and rsD_t_7:25964783) reached genome-wide significance after a Bonferroni correction for multiple testing (−log_10_ (*p*) ≥ 6.21), and these loci explained 10.10%, 9.31%, 9.18% and 8.95% of the phenotypic variation in FL, respectively (Fig. 3a, Table 1). For FS, five regions of association were identified. In these regions, ten SNP loci exhibiting associations with FS were distributed on chromosomes A_t_4, A_t_5, D_t_1, D_t_4 and D_t_7. For example, a SNP locus (rsD_t_7:27437213) on chromosome D_t_7 showed significant marker-trait associations with −log_10_ (*p*) values as high as 6.24, which explained 8.60% of the total observed variation in FS (Fig. 3b, Table 1). Moreover, five regions of association with FU were detected; however, they exhibited lower −log_10_ (*p*) values. Synthetically, the SNP locus D_t_7: 27437213 was significantly associated with FL and FS. Most importantly, all nine called SNP markers of the 66.77 kb region (D_t_7:25931988-25999761) demonstrated associations with FL.

### Two major genomic regions (MGR1 and MGR2) on chromosome D_t_7 and candidate genes potentially underlying FL and FS

**Figure 4:**
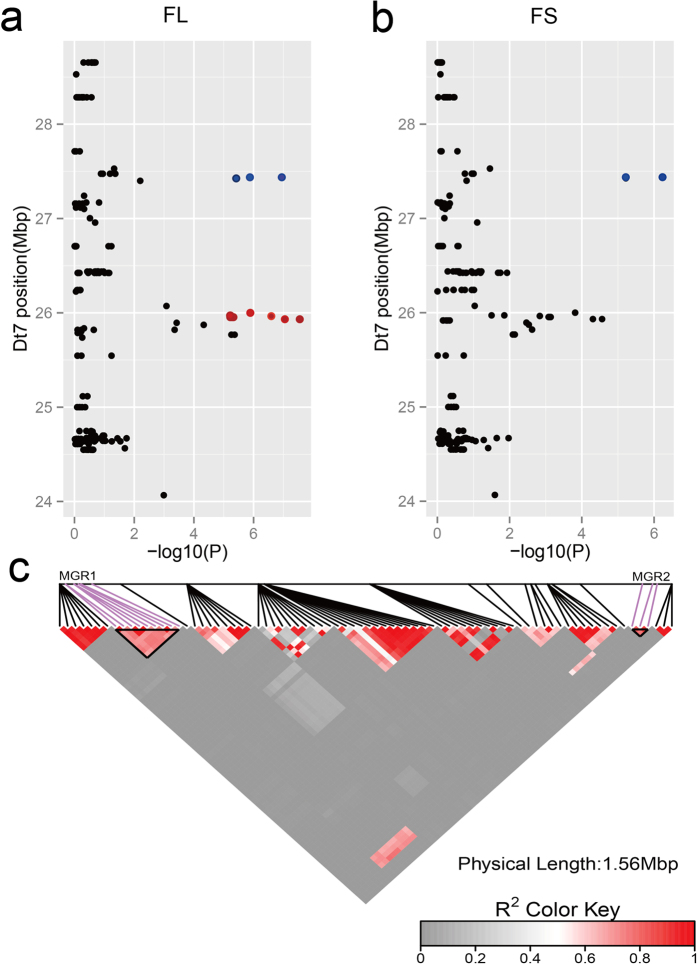
LD blocks of two major genomic regions (MGR1 and MGR2) on D_t_7 (**a**) and (**b**). Association signals of FL and FS in the region (24.00–29.00 Mb) on chromosome D_t_7. The red and blue dots represent SNPs contained in MGR1 and MGR2 in (**a**) and (**b**), respectively. (**c**) The distribution of LD blocks of two major genomic regions (MGR1 and MGR2) on D_t_7. The pair-wise LDs between the SNP markers are indicated as D’ values, where dark red indicates a value of 1 and gray indicates 0. The black triangles indicate LD blocks that contain significant SNPs.

**Figure 5:**
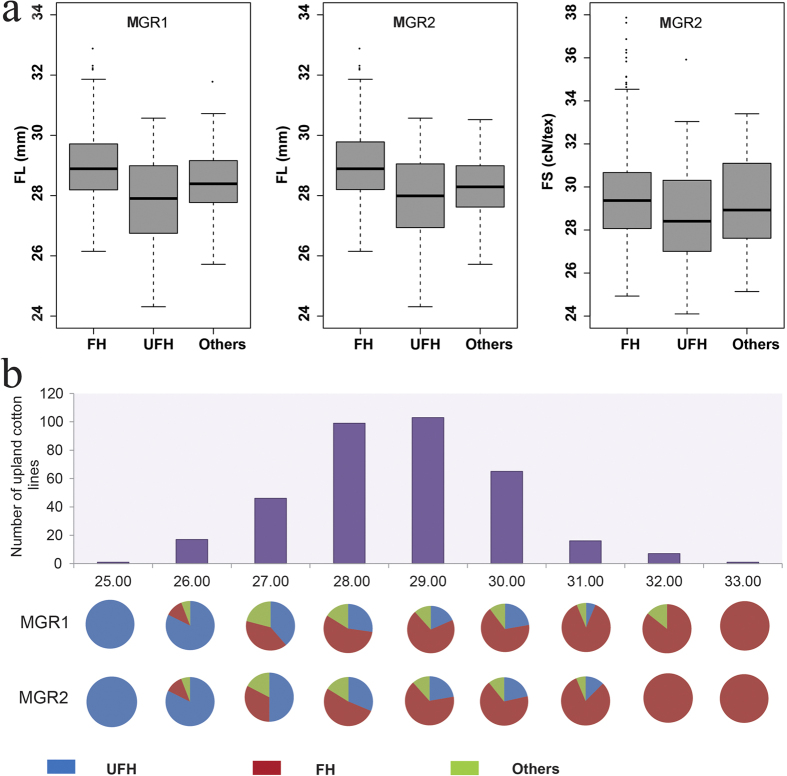
Phenotypic distributions for the haplotypes of the two major genomic regions (MGR1 and MGR2). (**a**) Box plots for phenotypic values of lines containing favorable haplotypes (FHs), unfavorable haplotypes (UFHs) and other haplotypes (Others). (**b**) Charts of the proportions of several types of haplotypes.

To identify putative candidate genes in the neighboring regions of the SNP loci associated with FL and FS, we further determined LD blocks harboring four significant SNPs (−log_10_ (*p*) > 6.21). The four SNPs were distributed in two LD blocks. Although the four significantly associated SNPs were contained in a smaller region of 1.51 megabase pairs (Mbp), they were distributed in two separate genomic regions distinguished by LD block analysis (Fig. 4). The first major genomic region of 66.77 kb (MGR1, D_t_7:25931988-25999761) consisted of nine SNP loci associated with FL on chromosome D_t_7 and was detected by GWAS. The nine SNP alleles were A/G, C/T, C/T, A/C, A/G, C/T, C/G, C/T and A/G, respectively. We observed a close linkage relation among the nine SNP loci associated with FL. The haplotype (AA-TT-CC-AA-AA-TT-GG-TT-AA) that included 188 lines was deemed the favorable haplotype (FH) because the mean FL (28.99 mm) of the haplotype was significantly higher than the mean FL (27.86 mm) of the other corresponding haplotype (GG-CC-TT-CC-GG-CC-CC-CC-GG; the unfavorable haplotype, UFH), which included 74 lines. The mean FL of the remaining 93 lines was 28.53 mm; in these lines, the number of lines containing 1–8 copies of the favored alleles was 6, 7, 9, 4, 4, 3, 8 and 12, respectively, and 40 lines included the haplotype (AG-CT-CT-AC-AG-CT-CG-CT-AG) (Fig. 5a). In addition, FH accounted for a large proportion of the upland cotton accessions with longer fibers, whereas UFH accounted for a larger proportion of the upland cotton lines with shorter fibers. For example, UFH was not observed in the lines with high fiber length (>31.50 mm), and FH was not observed in the lines with short fiber length (<25.50 mm) (Fig. 5b). The aforementioned results indicated that there might be a major gene controlling FL in MGR1 or an adjacent region. To search for putative candidate genes in MGR1 with the nine SNP loci associated with FL, three genes (*CotAD_22823, CotAD_22824* and *CotAD_22825*) have been annotated within the 66.77 kbp region of MGR1. *CotAD_22823* and *CotAD_22825* lack a definite annotation concerning their biological function. *CotAD_22823* contains two conserved domains of unknown function (DUF4013 and DUF3816), whereas *CotAD_22824* has a B3 binding domain, suggesting that it could be a member of the AP2/B3-like transcription factor family. Interestingly, the first and the second peak SNPs (rsD_t_7:25931998 and rsD_t_7:25932026) that were significantly associated with FL were positioned within one of the introns of *CotAD_22823*.

The second major genomic region of 11.74 kb (MGR2, D_t_7: 27425475-27437213) on chromosome D_t_7 includes three SNP alleles. Of the three SNPs, two (rsD_t_7:27436981 and rsD_t_7:27437213) had significant associations with both FL and FS, and their alleles were A/G and C/T, respectively. The FL and FS value of accessions with FH (GG-CC) in MGR2 were higher on average than those of accessions with UFH (AA-TT) (Fig. 5a). Similarly, FH accounted for a large proportion of the upland cotton accessions with longer fibers, whereas UFH accounted for a larger proportion of the upland cotton lines with shorter fibers (Fig. 5b). Interestingly, in MGR2, a peak SNP locus (D_t_7:27437213) associated with FS and its adjacent SNP locus (D_t_7:27436981) was distributed in the internal sequence of the gene *CotAD_35088*, and these results suggest that *CotAD_35088* is a candidate gene controlling FL and FS. *CotAD_35088* possesses a domain called a pentatricopeptide repeat (PPR) motif. The PPR protein gene family is distributed widely among terrestrial plants and has been shown to play an important role in plant development, organelle biogenesis, and cytoplasmic male sterility restoration.

### Geographic distribution and selection sweeps of favorable haplotypes for MGR1 and MGR2

**Figure 6:**
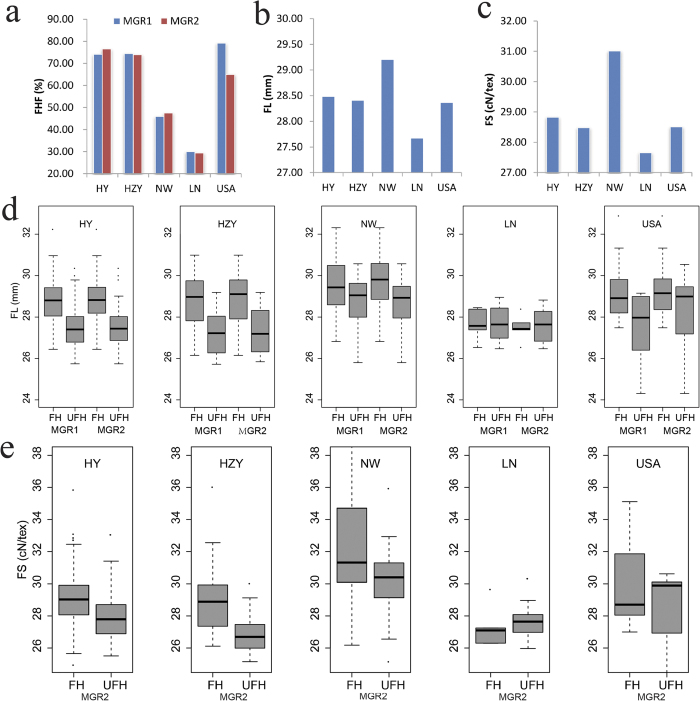
Geographic distribution and phenotypic values of the favorable haplotypes (FHs). (**a**) The favorable haplotype frequencies (FHFs) in the five geographic areas. (**b** and **c**) Phenotypic values of FL and FS in the five geographic areas. (**d**) Distribution of box plots for FL of the five geographic areas between the favorable haplotypes (FHs) and the unfavorable haplotypes (UFHs) in MGR1 and MGR2. (**e**) Distribution of box plots for FL of the five geographic areas between the favorable haplotype (FH) and the unfavorable haplotype (UFH) in MGR2.

To gain insight into the geographic distribution of two FHs in MGR1 and MGR2, a total of 355 upland cotton accessions from different ecological areas were divided into five groups: the YR group (162 accessions from the Yellow River region in China); YZR group (51 accessions from the Yangtze River region in China); NW group (98 accessions from the northwest inland region in China); LN group (20 accessions from Liaoning province in China); and USA group (20 accessions from the Texas cotton region of the United States of America). We analyzed the geographical distribution of two FHs among these upland cotton accessions, and heterozygous accessions were excluded from further analyses. Although the YR, YZR and USA lines had nearly the same level of FHF and showed little genetic differentiation, distinct FH distributions among the five different-source groups were found in MGR1 and MGR2 (Fig. 6a). For instance, there was a high FHF (>65%) in the lines obtained from YR, YZR and USA and a low FHF (<45%) in the accessions from NW and LN. Furthermore, we also found that the FHF of varieties from South Xinjiang (SXJ) were higher than those of varieties from North Xinjiang (NXJ) (Supplementary Fig. S2). This result suggests that the two FHFs were higher in accessions from low-latitude regions than in those from high-latitude regions in China. Surprisingly, the highest FL and FS were found in ecological areas, although the FHFs were lower in accessions from NW (Fig. 6b,c). We speculate that there might be other more important loci or genes controlling FL and FS in the accessions in the NW group. To identify the associated SNP loci in the NW accessions, another association analysis was conducted for the FL and FS traits using 98 NW upland cotton accessions. A towering distribution of SNP loci associated with FL and FS was found on chromosome A_t_4, and two SNP loci associated with target traits were detected (Supplementary Fig. S2). The results of the association analysis of upland cotton accessions from the NW region confirmed our tentative inference. In addition, a comparison of fiber quality between the FH and UFH groups indicated that the FL and FS of the FHs in the lines from YR, YZR, NW and USA were dramatically higher than those of the UFHs, whereas the FL and FS of the FHs in the lines from LN were not strikingly higher than those of the UFHs (Fig. 6d,e).

**Figure 7:**
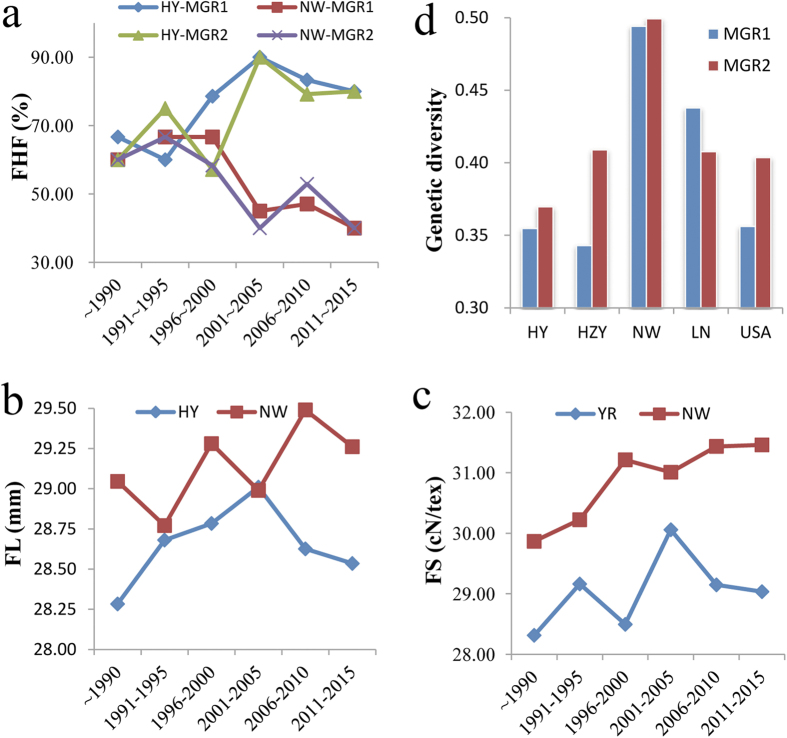
Differentiation of breeding period and genetic diversity in the two favorable haplotype frequencies (FHFs). (**a**) Breeding period differentiation of the two favorable haplotype frequencies (FHFs). (**b** and **c**) Breeding period differentiation of FL and FS. (**d**) Differentiation of genetic diversity of the five geographic areas in the two major genomic regions (MGR1 and MGR2).

Because of the FHF differences among the five geographic populations, the SNPs within the MGR1 and MGR2 regions may represent targets of artificial selection. To identify further potential selective sweeps of two FHs, a total of 77 (YR) and 79 (NW) source-identified and incubation-time-clear varieties were selected, and the FHF differentiation between MGR1 and MGR2 was scanned among varieties during six different breeding periods. We found that the FHFs of MGR1 and MGR2 differed among cultivars developed during different breeding periods. For cultivars developed before 2000, the FHFs did not exhibit obvious differences between YR and NW. However, the YR FHFs were much higher than those of NW for cultivars developed after 2000 because the FHFs resulted in a significant increase in YR and an evident reduction in NW between 2001 and 2005 (Fig. 7a). Afterwards, the FHFs of YR and NW exhibited a gradual decline. Interestingly, the coincidence of change trends in the FHFs and the FL and FS phenotypes were characterized, especially for YR lines (Fig. 7a–c). These results not only offer proof of target of selection within the MGR1 and MGR2 but also provide additional evidence of MGR1 and MGR2 control of FL and FS. Furthermore, we deduced that the FHs have strengthened the progress of artificial selection in upland cotton breeding over recent decades in the YR region of China.

To seek further evidence that the FHs have undergone selection, we analyzed the nucleotide diversity of the upland cotton population and found that the average genetic diversity values of the whole genome and chromosome D_t_7 were as high as 0.3730 and 0.3927, respectively, for all lines. However, the SNPs located within the MGR1 and MGR2 presented lower genetic diversity (Table S4). In MGR1, the genetic diversity of the germplasms containing FH ranged from 0 to 0.1095 and had an average of 0.0769, whereas the diversity of the germplasms containing UFH ranged from 0.1327 to 0.1984 and had a mean of 0.1448. However, when we analyzed the genetic diversity of the whole D_t_7 chromosome, we found that diversity was not significantly different between the FHs (0.3834) and UFHs (0.3890). In the MGR2, the genetic diversity of the accessions that included FHs exhibited an average value of 0.0966, and the diversity of accessions that included UFHs had a mean of 0.1407. Furthermore, we analyzed the difference in genetic diversity among eleven SNPs within the MGR1 and MGR2 for the five geographical areas and found that the genetic diversity of the YR and YZR lines were lower than that of the NW accessions (Fig. 7d). Therefore, we concluded that the low genetic diversity of FHs in HY and HZY is likely to be the result of selection pressure.

## Discussion

### Major genomic regions and QTL control FL and FS

QTL mapping is an important tool used by breeders to combine economically important traits to create a superior cultivar. A meta-QTL analysis of cotton based on 42 different studies was performed, and a total of 728 QTLs for fiber quality traits were mapped^3^. Over the past 15 years, a large number of QTL mapping studies have clearly indicated that a greater number of QTLs that control fiber quality traits are located on the D subgenome than on the A subgenome^31^,^32^,^33^,^34^,^35^. In this study, 25/33 (75.76%) SNP loci associated with fiber quality traits were distributed on the D subgenome, and two major genomic regions (MGR1 and MGR2) associated with FL were located on chromosome D_t_7. Therefore, our results are consistent with the opinion that the D subgenome provides a greater contribution to the genetic control of fiber quality traits than the A subgenome.

In previous studies, a large number of association analyses of fiber quality traits and SSR markers in upland cotton have been reported^13^,^14^,^36^,^37^. However, these studies were limited by the number of the polymorphic SSR markers and the size of the natural populations. Currently, a major concern is the requirement of high throughput genotyping and reliable phenotype identification. With the rapid development of sequencing technologies and computational methods, GWASs have become a powerful tool for detecting natural variation, genomic regions or candidate genes underlying elite traits in crops^38^. In the present study, two major genomic regions (MGR1 and MGR2) were found to be associated with FL; in particular, MGR1 contains nine SNP loci that are significantly associated with FL. Manhattan plots for FL typically indicated a towering distribution of many SNPs in one region of the genome, which indicated the dependability of the GWAS results. However, because of the low coverage of SLAF-sequencing, a towering distribution was found only for chromosome D_t_7, and a small number of SNP loci associated with target traits was detected. Thus, to increase the density of molecular markers, it is necessary to perform high-coverage whole genome sequencing for GWAS using large-scale populations. Despite these difficulties, we achieved the desired result. For example, four SNP loci associated with target traits (rsD_t_7:25931988, rsD_t_7:25932026, rsD_t_7:27436981 and rsD_t_7:27437213) were distributed within the sequences of two genes (*CotAD_22823* and *CotAD_35088*). These findings suggest that two major genomic regions and candidate genes for targeted traits represent effective targets for improving fiber quality in future cotton breeding.

**Figure 8:**
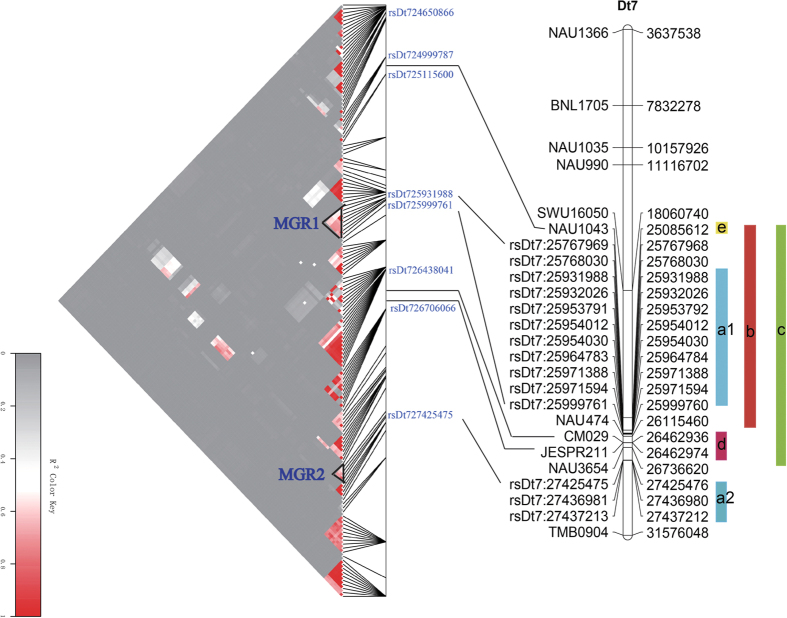
Comparison of the GWAS with QTLs identified in previous studies. The left-hand chart represents the LD blocks of two major genomic regions (MGR1 and MGR2) on D_t_7; the right-hand chart represents a physical map of chromosome D_t_7 containing molecular markers from our GWAS and QTL and association mapping from previous studies. a1 and a2 represent MGR1 and MGR2, respectively, including the SNP loci in our GWAS. b, c and d represent QTL mapping from previous studies; the intervals b, c, and d represent qFL-7-1a (NAU1043-NAU474)^34^, qFS-LG05-1 (NAU1043-NAU3654)^41^ and a QTL (JESPR211-CM029)^42^; e represents an SSR marker associated with FL from association mapping of previous studies.

To fully understand the behavior of complex traits, the new GWAS must be compared with previous linkage and association studies. In previous QTL mapping studies, 557 SSR markers (Tables S5–S6) containing QTLs of FL and FS from 34 reports of QTL mapping were selected (Table S7), and 268 primer sequences corresponding to these markers (Table S5) were gained from the CottonGen Database (http://www.cottongen.org). The physical locations of these SSR primer sequences were mapped to the reference genome sequence^39^ by electronic PCR (e-PCR). In previous studies, at least 11 SSR markers were mapped to chromosome D_t_7 (Fig. 8). One SSR marker, NAU1043, mapped to D_t_7 has been reported in many studies. For example, Yu *et al*.^40^, Shen *et al*.^34^, Wang *et al*.^41^ and Cai *et al*.^14^ reported FL or FS QTLs linked to the NAU1043 marker. Interestingly, MGR1, including the nine SNP loci in our study, is distributed in QTLs identified in previous studies, such as qFL-7-1a (NAU1043-NAU474)^34^ and qFS-LG05-1 (NAU1043-NAU3654)^41^. Wu *et al*.^42^ also identified a QTL (JESPR211-CM029) for FL in an adjacent region of MGR1 and MGR2. These findings validate the GWAS results and increase confidence in the identity of some SNP loci of MGR1. To determine the LD extent between our GWAS results and the QTL intervals or loci from previous studies, LD blocks harboring significantly associated SNPs and their neighboring SNPs on chromosome D_t_7 were defined. The results indicated low levels of LD between NAU1043 and MGR1, between d and MGR1 and between d and MGR2 (Fig. 8). These findings indicated that there were different genome regions between the GWAS results (MGR1 and MGR2) and the QTL intervals from previous studies. In addition, SNP markers near the physical locations of the 268 SSR primers and their corresponding *p* values were screened out, and a total of eleven SSR markers near the SNP loci with −log_10_ (*p*) > 2.0 were detected (Tables S8 and S9). For example, NAU474 was closest to the SNP locus rsD_t_7:26072147, with a larger −log_10_ (*p*) value of 3.05 associated with FL. Although a comparison of the new GWAS with QTLs identified in previous studies was performed, it is very difficult to compare different QTLs for FL and FS in various populations, particularly in a changing environment. The vast majority of QTLs based on SSR markers for fiber quality traits have been mapped by crossing populations between *G. hirsutum* and *G. barbadense*, whereas the SNP loci associated with FL and FS were identified via GWAS in a natural population of *G. hirsutum* in the present study. Furthermore, only a rough draft of the upland cotton reference genome sequence is available, hindering highly accurate physical location of each marker. Hence, it was not possible to precisely integrate all the SSR and SNP markers into the reference genome sequence, and many of the QTLs differed between our study and previous studies.

Two sets of upland cotton (TM-1) reference genome sequences^39^,^43^ have been completed, and the chromosome numbering of these genome sequences differs. To correspond to the chromosomal location of the SNP loci associated with the target traits, the genome sequences of MGR1 and MGR2 were extracted from the upland cotton reference genome^39^ and aligned with the other upland cotton reference genome^43^. The chromosome corresponding to D_t_7 in the other upland cotton reference genome is D11, and D_t_7 corresponds to C21 in the linkage groups. The chromosomal position of each associated SNP locus was also determined for a second reference genome (Table 2). A meta-QTL analysis showed that C21 contains six and five QTLs for FL and FS, respectively^3^. Moreover, several QTLs for FL and FS mapped to C21 (D_t_7)^10^,^35^. The QTL cluster for FL and FS on C16 has been observed in previous studies^3^,^35^,^44^. However, the SNP loci that were significantly associated with FL and FS were not detected for C16 in this study, most likely for the following reasons: (1) fewer SNP markers with low coverage were distributed on C16; or (2) a large number of QTLs for fiber quality were mapped using linkage mapping methods and interspecific-crossing populations between *G. hirsutum* and *G. barbadense* in recent decades. Therefore, it is important to develop additional markers for use in future studies.

### Geographic distribution and selection sweeps of FHs

To identify and access the allelic variations affecting crop phenotypes, it is important to comprehensively evaluate and characterize large-scale representative genetic resources. In recent years, numerous studies have used a large number of germplasms and performed selective sweeps related to the domestication and improvement of crop traits. For example, the coincidence of salt-affected soils and salt-tolerant haplotypes in soybean indicates that these alleles are likely to be a major selection factor determining the distribution and utilization of soybean, particularly in saline soils^45^. In maize, thousands of genomic regions have been associated with artificial selection targets during modern breeding and domestication^26^,^46^, with certain genes in these regions representing key factors that control traits that have been improved in recent decades^46^. Similar studies have also been conducted for wheat^23^, soybean^28^, rice^25^, tomato^47^ and rapeseed^48^. In this study, a GWAS of large-scale upland cotton populations was able to successfully authenticate the selective signals related to domestication and fiber quality trait improvements for two major genomic regions (MGR1 and MGR2), which will aid future improvement of fiber quality and the identification of new domestication genes.

Composite likelihood ratios (CLRs) and F_ST_ values are commonly used in the identification of genomic targets of artificial selection^49^,^50^, and genomic regions with extreme allele frequency differentiation have been detected. The strength and duration of selection can impact the frequency and distribution of the selected alleles among individual populations^23^. Geographic patterns of genetic differentiation have long been used to determine the population history and the biological mechanisms of adaptation for different organisms^51^. For example, an examination of the genomic patterns of differentiation between northern and southern populations of Australian and North American *Drosophila simulans* has provided insight into common selective pressures and responses^51^. In our study, selective sweeps of two major genomic regions were examined by a conventional statistical approach that compared FHF, and we found that the FHFs of MGR1 and MGR2 had distinct haplotype distributions in varieties obtained from different eco-regions in China. Further investigation indicated that the FHFs of the low-latitude regions were strikingly higher than that of the high-latitude regions in China. Moreover, these results showed that the FHFs and fiber length in NXJ were lower than the FHFs and fiber length in SXJ. These distinct patterns of geographic distribution among haplotypes subjected to selection could be associated with adaptations to local environmental conditions. Because the high-latitude regions (e.g., NW and LN) experience a shorter growth duration and lower accumulated temperature in China, the prematurity of upland cotton was considered the most important breeding objective. Breeders seeking to achieve early maturation have ascribed little importance to the FHs for fiber quality because of the negative genetic correlation between fiber quality and earliness. However, in YR and YZR, which experience a later harvest time, the FHs for fiber quality have been selected artificially by many breeders, and the target traits were improved in these cultivars. The FH distributions of MGR1 and MGR2 among different populations constitute a valuable resource that can be used to design future breeding strategies. In addition, the frequency differentiation of favorable alleles for cotton lint yield components in historically released cultivar groups has been reported in a previous study^52^. Another study reported that elite QTL alleles for fiber quality traits in the three breeding periods were passed down from the four core cultivars, whereas other QTL alleles detected in the core cultivars were not selected by breeders in the development of modern Chinese cotton cultivars^13^. In this study, a considerable difference in FHFs was observed between YR and NW cultivars developed after 2000, which was most likely because the FHs with high strength were selected and passed down from early-period to late-period cultivars in YR. These results also indicate that FHs have experienced artificial selection during upland cotton breeding in China.

The detection of genome-wide genetic diversity and the identification of candidate genes that contribute to the domestication and improvement of target traits are essential for breeding superior varieties^46^,^53^. Prior studies of upland cotton found that the genetic diversity in varieties from the YZR and YR regions was higher than that of NW varieties^14^. However, we observed that the genetic diversity of YR, YZR and USA lines was lower than that of NW and LN accessions for two major genomic regions (MGR1 and MGR2). In summary, the high FHF and the low genetic diversity of plants obtained from lower latitude areas in China are hallmarks that signal strongly favored haplotypes during natural and artificial selection.

## Methods

### Plant materials and phenotyping

A total of 355 upland cotton accessions (Table S10) obtained from the cotton germplasm collections in our laboratory and the low-temperature germplasm gene bank of the Cotton Research Institute of the Chinese Academy of Agricultural Sciences (CRI-CAAS), were planted in a randomized complete block design with three replications at two locations in Anyang (AY), Henan (36°08′N, 114°48′E) and Shihezi (SHZ), Xinjiang (44°31′N, 86°01′E) over two years (2014 and 2015). Twenty normally opened bolls from middle fruiting branches of each replicate were sampled annually in September. Fiber samples weighing 10–15 g were measured using an HVI-MF 100 instrument (User Technologies, Inc., USTER, Switzerland) at the Cotton Fiber Quality Inspection and Testing Center of the Ministry of Agriculture, Anyang, China. The following fiber quality traits were evaluated: 50% fiber span length (FL, mm), fiber strength (FS, cN/tex), fiber uniformity (FU, %), fiber micronaire (FM) and fiber elongation (FE, %).

### Genotyping by SLAF-sequencing

A total of 81,675 SNP markers were used for the subsequent analysis. SNP genotyping was performed using an SLAF-seq approach^54^. Two restriction enzymes (*Rsa* I and *Hae* III, New England Biolabs, NE, USA) were used for library preparation. Paired-end sequencing (80 bp at each end) was performed on an Illumina HiSeq 2500 system (Illumina, Inc., San Diego, CA, USA) according to the manufacturer’s recommendations. The GATK and SAMtools packages were used for SNP calling, and BWA software was used to map the raw paired-end reads onto the reference genome (*Gossypium hirsutum* v 1.0)^39^.

### Genetic diversity and population structure analysis

The geographic location of each upland cotton accession was obtained from the CRI-CAAS database. A map of the geographic positions of 331 accessions gathered from China was generated using the R software package ‘maptools’ (http://r-forge.r-project.org/projects/maptools/) and is shown in Fig. 2a. Power-Marker v 3.25^53^ software was used to estimate the genetic diversity of SNP markers for the tested cotton accessions. The genetic diversity values of each of group were calculated according to the chromosome. Nei’s^55^ genetic distances among the 355 upland cotton accessions were calculated, and a neighbor-joining dendrogram was constructed with Power-Marker V 3.25 software.

The structure of the natural upland cotton population was analyzed using a PCA approach with the GAPIT software package^56^.

### Genome-wide association studies

The best linear unbiased prediction (BLUP) values of five fiber quality traits in four environments were estimated using the R software package ‘lme4’^57^. PCA was superior to the Q model in controlling false positives for the estimation of population structure^58^,^59^. Therefore, a mixed linear model (MLM) was used to calculate the associations in all analyses by incorporating PCA and kinship data^56^. The suggestive and significant *p* thresholds were 6.12E–06 and 6.12E–07 for the entire population, respectively^60^,^61^. Manhattan plots were generated using the R software package ‘CMplot’.

### Haplotype analysis

The phenotypic value of each haplotype was estimated through the average phenotypic value over accessions for each type of SNP locus associated with the target trait. The FHs were subsequently identified according to the breeding objective of each target trait. Box plots of the relative phenotypic values were generated using R software. The FHFs of the SNP loci associated with FL and FS were calculated via statistical methods using R software.

## Additional Information

**Accession codes**: The sequence read data from the SLAF-seq analysis of 355 sequenced upland cotton lines have been submitted and are available at the Sequence Read Archive (http://www.ncbi.nlm.nih.gov/ bioproject/PRJNA314284/SRP071133) under the accession number PRJNA314284.

**How to cite this article**: Su, J. *et al*. Two genomic regions associated with fiber quality traits in Chinese upland cotton under apparent breeding selection. *Sci. Rep.* **6**, 38496; doi: 10.1038/srep38496 (2016).

**Publisher’s note:** Springer Nature remains neutral with regard to jurisdictional claims in published maps and institutional affiliations.

## Supplementary Material

Supplementary Information

## Figures and Tables

**Table 1:** Details of loci associated with fiber quality traits identified via genome-wide association studies (GWASs) in upland cotton.

Traits	Regions	SNP loci	Chromosome	Position (Mbp)	−log_10_(*p*)	R^2^ (%)	Genes^a^
FL	1	rsA_t_9:31687000	A_t_9	31.69	5.21	6.95	
		rsA_t_9:31778023	A_t_9	31.78	5.23	6.91	
	2	rsD_t_7:25767969	D_t_7	25.77	5.26	6.94	
		rsD_t_7:25768030	D_t_7	25.77	5.36	7.07	
	3 (MGR1)	rsD_t_7:25931988	D_t_7	25.93	7.5	10.10	*CotAD_22823*
		rsD_t_7:2593026	D_t_7	25.93	7.05	9.31	*CotAD_22823*
		rsD_t_7:25953791	D_t_7	25.95	5.32	7.30	
		rsD_t_7:25954012	D_t_7	25.95	5.28	7.25	
		rsD_t_7:25954030	D_t_7	25.95	5.22	7.04	
		rsD_t_7:25964783	D_t_7	25.96	6.59	8.95	
		rsD_t_7:25971388	D_t_7	25.97	5.22	7.12	
		rsD_t_7:25971594	D_t_7	25.97	5.21	6.74	
		rsD_t_7:25999761	D_t_7	26.00	5.89	8.00	
	4 (MGR2)	rsD_t_7:27425475	D_t_7	27.43	5.42	7.49	
		rsD_t_7:27436981	D_t_7	27.44	5.88	7.12	*CotAD_35088*
		rsD_t_7:27437213	D_t_7	27.44	6.95	9.18	*CotAD_35088*
FS	1	rsA_t_4:69980131	D_t_7	69.98	5.43	7.49	
		rsA_t_4:69980135	D_t_7	69.98	5.65	7.74	
	2	rsA_t_5:17565858	A_t_5	17.57	5.26	7.09	
		rsD_t_1:101881672	D_t_1	101.88	5.33	6.93	
		rsD_t_1:101881897	D_t_1	101.88	5.44	7.07	
	4	rsD_t_1:102969462	D_t_1	102.97	5.20	86	
		rsD_t_1:102969650	D_t_1	102.97	5.35	6.10	
	5	rsD_t_4:8362683	D_t_4	8.36	5.21	6.75	
	6 (MGR2)	rsD_t_7:27436981	D_t_7	27.44	5.22	6.02	*CotAD_35088*
		rsD_t_7:27437213	D_t_7	27.44	6.24	8.60	*CotAD_35088*
FU	1	rsA_t_6:10202790	A_t_6	10.20	5.46	7.33	
	2	rsA_t_91687000	A_t_9	31.69	5.06	6.96	
		rsA_t_9:31778023	A_t_9	31.78	5.22	6.53	
	3	rsD_t_3:4260283	D_t_3	4.26	5.23	6.72	
		rsD_t_3:4706150	D_t_3	4.71	5.39	6.33	
	4	rsD_t_5:40993634	D_t_5	40.99	5.24	7.03	
		rsD_t_5:4093882	D_t_5	40.99	5.25	7.01	

^a^Genes are annotated according to Li *et al*.^38^; the associated SNP loci were positioned within the gene sequence of genes.

MGR1: first major genomic region; MGR2: second major genomic region.

**Table 2:** Comparison of the chromosomal positions of the SNP loci and candidate genes potentially underlying fiber length and strength between the two upland cotton reference genomes.

Major regions	Traits	Chromosome^a^	Site^a^	Chromosome^b^	Site^b^	Linkage group
MGR1	FL	D_t_7	25931988	D11	24034609	C21
		D_t_7	25932026	D11	2403460	C21
		D_t_7	25953791	D11	24056372	C21
		D_t_7	25954012	/	/	C21
		D_t_7	25954030	D11	4056611	C21
		D_t_7	25964783	D11	24067326	C21
		D_t_7	25971388	D11	24073931	C21
		D_t_7	25971594	D11	24074137	C21
		D_t_7	25999761	D1	24102240	C21
MGR2	FL	D_t_7	7436981	scaffold4548_D11	25364	C21
	FS	D_t_7	27437213	scaffold4548_D11	25132	C21

^a^Upland cotton reference genome according to Li *et al*.^38^.

^b^Upland cotton reference genome according to Zhang *et al*.^42^.
