# Supplementary material for: Two genomic regions associated with fiber quality traits in Chinese upland cotton under apparent breeding selection
Source: Sci Rep. 2016 Dec 7;6:38496. doi: 10.1038/srep38496 (PMC5141495; doi:10.1038/srep38496)
Supplement: Supplementary Information [file srep38496-s1.pdf]

Two genomic regions associated with fiber quality traits in Chinese upland cotton under apparent breeding selection

Junji Su[1,2], Libei Li[1], Chaoyou Pang[1], Hengling Wei[1], Caixiang Wang[1], Meizhen Song[1], Hantao Wang[1], Shuqi Zhao[1], Chi Zhang[1], Guangzhi Mao[1], Long Huang[3], Chengshe Wang[2], Shuli Fan[1*], Shuxun Yu[1,2*]

[1] State Key Laboratory of Cotton Biology, Institute of Cotton Research of CAAS, 455000, Anyang, China

[2] College of Agronomy, Northwest A&F University, 712100 Yangling, China

[3] Bioinformatics Division, Biomarker Technologies Corporation, 101300 Beijing, China

Correspondence to S.L.F. (email: fsl427@126.com) or S.X.Y. (email: ysx195311@163.com)

Supporting information

Additional supporting information may be found in the online version of this article.

Table S1. Descriptive statistics of the fiber quality traits in four different environments.

Table S2. Correlations among the five fiber quality traits based on the 355 upland cotton accessions.

Table S3. Genetic diversity of the natural population.

Table S4. Genetic diversity of the haplotypes of the two major genomic regions.

Table S5. 268 SSR primers with sequence obtained for public databases.

Table S6. 289 SSR primers without sequence obtained for public databases

Table S7. The QTLs of FL and FS from 34 reports of QTL mapping

Table S8. SNP markers near physical locations of 268 SSR markers and their corresponding p value associated with FL

Table S9. SNP markers near physical locations of 268 SSR markers and their corresponding p value associated with FS

Table S10. Information on 355 upland cotton germplasms.

Supplementary Fig. S1. Geographic distribution and phenotypic values of the favorable haplotypes (FHs) in Xinjiang, China.

Supplementary Fig. S2. Manhattan plots of the genome-wide association studies (GWASs) of NW upland cotton accessions for fiber length and strength.

Table S1 Descriptive statistics of the fiber quality traits in four different environments

Traits	FL (mm)	FU (%)	FM	FE (%)	FS
MIN	23.25	79.40	2.52	6.03	22.70
MAX	34.59	87.17	6.00	7.20	40.65
MEAN	28.64	84.32	4.78	6.65	29.33
CV (%)	5.01	1.43	9.06	1.60	8.82

Table S2 Correlations among the five fiber quality traits based on the 355 upland cotton accessions

Traits	FL	FU	FM	FE	FS
FL	1.0000				
FU	0.7592	1.0000			
FM	-0.3554	-0.0151	1.0000		
FE	0.7439	0.6770	-0.1804	1.0000	
FS	0.8639	0.6930	-0.3736	0.6694	1.0000

Table S3 Genetic diversity of the natural populations

Chromosome	Gene Diversity	Chromosome	Gene Diversity
At1	0.3586	Dt1	0.3829
At2	0.3747	Dt2	0.3723
At3	0.3539	Dt3	0.3825
At4	0.3625	Dt4	0.3465
At5	0.3597	Dt5	0.4056
At6	0.3565	Dt6	0.3741
At7	0.3816	Dt7	0.3927
At8	0.3603	Dt8	0.3829
At9	0.3856	Dt9	0.3829
At10	0.3485	Dt10	0.3487
At11	0.3541	Dt11	0.3724
At12	0.3677	Dt12	0.4013
At13	0.3897	Dt13	0.3897
Mean	0.3656		0.3796

Table S4 Genetic diversity of the haplotypes of the two major genomic regions

Major regions	SNP Loci	Gene Diversity	
		FH	UFH
MGR1	rsDt725931988	0.0000	0.1434
	rsDt725932026	0.0000	0.1438
	rsDt725953791	0.1095	0.1408
	rsDt725954012	0.1049	0.1408
	rsDt725954030	0.0971	0.1420
	rsDt725964783	0.1074	0.1486
	rsDt725971388	0.0790	0.1327
	rsDt725971594	0.0849	0.1132
	rsDt725999761	0.1089	0.1984
Mean		0.0769	0.1448
MGR2	rsDt727436981	0.0918	0.1502
	rsDt727437213	0.1014	0.1312
Mean		0.0966	0.1407

Table S5 268 SSR primers with sequence obtained from public databases

N0.	Primers	N0.	Primers	N0.	Primers	N0.	Primers	N0.	Primers
1	BNL1414	41	NAU2126	81	NAU4034	121	Gh397	161	BNL2960
2	HAU0886	42	NAU5091	82	NAU2035	122	JESPR114	162	NAU3967
3	BNL1317	43	GH663	83	JESPR215	123	JESPR127	163	BNL1231
4	NAU3145	44	NAU3541	84	JESPR220	124	NAU2665	164	BNL2662
5	MON_DC40182	45	CIR328	85	NAU2631	125	BNL3280	165	NAU3071
6	CIR081	46	CIR216	86	BNL119	126	SWU19198	166	NAU5379
7	BNL1434	47	NAU943	87	JESPR208	127	NAU2627	167	NAU2730
8	MON_CGR5040	48	CIR099	88	SWU14535	128	NAU4951	168	BNL3627
9	BNL3145	49	MUSB0979	89	SWU10214	129	SWU16050	169	CIR238
10	TMB0515	50	NAU3273	90	HAU3186	130	BNL1705	170	CIR213
11	NAU2297	51	SWU19175	91	NAU3917	131	MON_CGR5145	171	NAU2865
12	BNL3558	52	NAU1200	92	PGML00353	132	NAU4064	172	TMB1296
13	SWU21453	53	NAU4099	93	BNL1694	133	NAU3254	173	NAU3769
14	BNL3474	54	NAU2926	94	CIR148	134	NAU2343	174	BNL4108
15	NAU3735	55	CIR354	95	CIR148	135	NAU5390	175	PGML00802
16	SWU16783	56	NAU3380	96	MUCS034	136	HAU1315	176	BNL2961
17	NAU3308	57	BNL3140	97	NAU1035	137	TMB2899	177	BNL2485
18	BNL3502	58	Gh617	98	NAU1322	138	NAU0902	178	BNL3650
19	NAU4057	59	NAU1037	99	NAU2684	139	SWU21550	179	NAU1336
20	NAU4024	60	HAU1316	100	DPL0067	140	NAU1048	180	BNL1521
21	NAU6173	61	BNL2530	101	SWU16034	141	Gh153	181	BNL1495
22	SWU15579	62	HAU035	102	GH222	142	BNL2568	182	TMB2295
23	NAU990	63	NAU3665	103	NAU2600	143	STV022	183	PGML00222
24	NAU3988	64	NAU3393	104	JESPR307	144	NAU3839	184	CIR078
25	HAU3213	65	GH388	105	BNL3580	145	BNL243	185	CIR077
26	NAU3605	66	NAU1366	106	NAU3212	146	DPL0395	186	HAU1974
27	NAU1092	67	BNL3279	107	MUSS193	147	NAU1043	187	MON_CGR5870
28	NAU1302	68	Gh060	108	BNL3410	148	GH120	188	CIR244
29	NAU3155	69	Gh268	109	NAU2277	149	SWU14514	189	NAU2395
30	CIR364	70	JESPR65	110	NAU5083	150	CIR105	190	NAU1369
31	NAU3823	71	GH144	111	HAU1455	151	MUSS280	191	NAU6642
32	MUCS135	72	BNL1440	112	HAU1603	152	DPL0570	192	HAU1044
33	NAU0828	73	CIR293	113	NAU2119	153	GH398	193	SWU15094
34	NAU3948	74	BNL4030	114	BNL3255	154	SWU14506	194	HAU2022
35	SWU14507	75	HAU1057	115	CIR307	155	SWU15397	195	SWU15599
36	NAU1197	76	NAU4045	116	NAU2581	156	Gh537	196	NAU5480
37	NAU2708	77	NAU3096	117	Gh058	157	NAU3713	197	NAU3654
38	BNL1454	78	NAU5399	118	JESPR297	158	TMB0904	198	CIR246
39	DPL0490	79	HAU2065	119	BNL4034	159	DPL0667	199	NAU3499
40	NAU1085	80	SWU21198	120	SWU14475	160	NAU2165	200	NAU3327

Table S5 268 SSR primers with sequence obtained from public databases (Continued)

N0.	Primers	N0.	Primers	N0.	Primers	N0.	Primers	N0.	Primers
201	B NL2920	215	BNL3034	229	BNL3482	243	NAU3117	257	BNL3431
202	BNL1605	216	MUSS151	230	NAU2302	244	NAU3390	258	BNL3029
203	NAU1295	217	NAU6136	231	NAU3464	245	BNL3090	259	BNL1122
204	CIR381	218	NAU1004	232	NAU3558	246	BNL3255	260	NAU3014
205	PGML00727	219	NBRI_Gh_B008	233	JESPR211	247	NAU4925	261	NAU3639
206	SWU19320	220	NAU5238	234	NAU1366	248	NAU6463	262	NAU1151
207	BNL1064	221	NAU933	235	NAU2121	249	NAU3608	263	BNL3452
208	BNL1030	222	NAU2712	236	NAU913	250	JESPR7	264	NAU5064
209	NAU3260	223	NAU4865	237	NAU1068	251	NAU2987	265	NAU1233
210	NAU3207	224	BNL3031	238	NAU972	252	NAU923	266	BNL2570
211	BNL3033	225	NAU3368	239	NAU2995	253	BNL3948	267	NAU5335
212	CM029	226	NAU2139	240	NAU3109	254	NAU1043	268	NAU474
213	NAU3324	227	NAU943	241	NAU1322	255	NAU1232		
214	NAU980	228	NAU3036	242	NAU3954	256	NAU5489		

Table S6 289 SSR primers without sequence obtained from public databases

N0.	Primers	N0.	Primers	N0.	Primers	N0.	Primers	N0.	Primers
1	A1152	41	CER168	81	DC40122a	121	E4M6d	161	MUSS145b
2	A1158b	42	CFB5875	82	DC40175	122	G1033	162	MUSS298b
3	A1172	43	CFB5877	83	DC40182	123	G1033a	163	N1
4	A1204	44	CFB5878	84	DC40429	124	G1104	164	NAU0895a
5	A1252	45	CGR5040	85	DOW006a	125	G1112a	165	NAU0913b
6	A1296	46	CGR5139	86	DPL0028b	126	G1125b	166	NAU1037b
7	A1310a	47	cgr5510	87	DPL0071a	127	G1147	167	NAU1043e
8	A1310b	48	cgr5565	88	DPL0080b	128	G1185a	168	NAU1356c
9	A1413	49	CGR5594	89	DPL0183a	129	GH329a	169	NAU1529
10	A1580	50	CGR5602	90	DPL0207	130	HAU0086b	170	NAU1534
11	A1672	51	CGR5620	91	DPL0236a	131	HAU0087b	171	NAU1595
12	A1686a	52	CGR5732	92	DPL0252	132	HAU1413a	172	NAU2083c
13	A1707a	53	CGR5873	93	DPL0284	133	HAU1417a	173	NAU2162a
14	A1727	54	CGR6012	94	DPL0375	134	HAU1417b	174	NAU2217a
15	AGC/CAC1	55	CGR6017	95	DPL0481a	135	HAU244	175	NAU2265a
16	AGG/CTC1	56	CGR6129	96	DPL050a	136	HAU3233a	176	NAU2291b
17	BNL0598	57	CGR6205a	97	DPL0532b	137	HAU880	177	NAU2477b
18	BNL0840a	58	CGR6217	98	DPL0562	138	HAU892	178	NAU2532
19	BNL1026a	59	CGR6218a	99	DPL0595	139	ICRI010391	179	NAU2560a
20	BNL1059a	60	CGR6508	100	DPL0600	140	IT-ISJ07F54R	180	NAU3201b
21	BNL1167a	61	CGR6528	101	DPL0615a	141	JESP295a	181	NAU3474b
22	BNL1227a	62	CGR6683	102	DPL077	142	JESPR006	182	NAU3822a
23	BNL140	63	CGR6784	103	DPL0852	143	JESPR019	183	NAU429
24	BNL1421a	64	CGR6802	104	DPL0852a	144	JESPR230a	184	NAU462
25	BNL1434a	65	CGR6817	105	DPL090	145	JESPR238a	185	NAU4956a
26	BNL1521a	66	CGR6902b	106	DPL0922	146	JESPR289a	186	NAU5046a
27	BNL1672a	67	CIR070a	107	DPL1016b	147	Lc1	187	NAU5099a
28	BNL2469	68	CIR195a	108	DPL1201a	148	m10e10-430	188	NAU5233
29	BNL2599a	69	CIR218a	109	DPL1379	149	m11e11-780	189	NAU5262a
30	BNL2611b	70	CIR381b	110	DPL146	150	M1E6	190	NAU6094a
31	BNL2733a	71	CM0027a	111	DPL182	151	m3e2-420	191	NAU6101
32	BNL3031b	72	CM0066a	112	DPL1931	152	M5E159.3	192	NAU6474b
33	BNL3259b	73	CM0160b	113	DPL286	153	m7e11-360	193	NAU6723a
34	BNL3400a	74	COT012	114	DPL303	154	m8e17-300	194	NAU6723b
35	BNL3594b	75	CRI100	115	DPL565	155	m8e17-700	195	NAU701
36	C2-0114	76	CSHE102a	116	DPL687	156	MGHES18	196	NAU733
37	C2-0114a	77	CSHE109	117	E17M8	157	MGHES58b	197	NAU780
38	CAC/TCA-312	78	CSHE51	118	E19M5	158	MON_DPL0405	198	NAU845
39	CER0168	79	CSHE99b	119	E2M7a	159	MUSB0846a	199	NL1017
40	CER028	80	CSHES150	120	E2M7f	160	MUSS106a	200	NL2634

Table S6 289 SSR primers without sequence obtained from public databases (Continued)

N0.	Primers	N0.	Primers	N0.	Primers	N0.	Primers	N0.	Primers
201	NL3140	221	pAR291a	241	pGH530	261	SNP0404	281	TMD05
202	NL3383	222	pAR3	242	pGH530new	262	SNP0464	282	TMHM8
203	OPal19s	223	pAR338a	243	pGH861	263	SSR152	283	TMK12
204	OPap01	224	pAR418a	244	PGML00463a	264	SSR1521	284	TMK19
205	OPap1	225	pAR4-34a	245	pVNC163a	265	SSR2961	285	TM006
206	OPm07	226	pAR864	246	SHIN-0272a	266	STV023a	286	ubc301
207	P2-9	227	pAR503	247	SHIN-0438a	267	SWU15058a	287	ubc431
208	P5	228	pAR547	248	SHIN-0966a	268	SWU16370b	288	ubc757
209	pAR038	229	pAR570a	249	SHIN-1131a	269	SWU17573b	289	UCD120b
210	pAR077a	230	pAR571b	250	SHIN-1138b	270	T1		
211	pAR101b	231	pAR788	251	SHIN-1344	271	T2E5c		
212	pAR137a	232	pAR792	252	SHIN1447	272	T3E8b		
213	pAR1-56	233	UCD216b	253	SHIN-1452a	273	T4E3a		
214	pAR168b	234	pGH225	254	SHIN-1542a	274	T5E4		
215	pAR172b	235	pGH243	255	SHIN-1547a	275	TCE6b		
216	pAR188	236	pGH286	256	SHIN-1552a	276	TM006		
217	pAR206a	237	pGH309	257	SNP0080	277	TMB0323a		
218	pAR209	238	pGH318b	258	SNP0189	278	TMB1208b		
219	pAR238	239	pGH399	259	SNP0227	279	TMB1222b		
220	pAR243	240	pGH468	260	SNP0256	280	TMC005a		

Table S7 The QTLs of FL and FS from 34 reports of QTL mapping

No. of reference	Traits	QTLs	Chro.	marker1	marker2	LOD	Additive
1	FL	qFL1-1	A1	NAU2083c	NAU1356c	2.53	1.39
		qFL21-1	D11	NBRI_Gh_B008	NAU5064	2.98	-1.56
		qFL14-1	D2	NAU3474b	NAU3464	2.54	1.42
		qFL7-1	A7	MGHES58b	NAU4956a	5.15	2.19
		qFL16-1	D7	NAU6136	STV023a	3.05	-1.15
		qFL16-2	D7	NAU6723a	TMHM8	3.62	-1.20
		qFL16-3	D7	NAU6723b	NAU5238	3.40	-1.89
		qFL22-1	D4	NAU913	CAC/TCA-312	4.63	2.38
	FS	qFS5-1	A5	NAU6094a	NAU2121	3.54	-2.26
		qFS22-1	D4	NAU2302	NAU2712	2.90	-1.92
		qFS3-2	A3	NAU1068	NAU972	4.50	2.54
		qFS3-1	A3	NAU972	NAU6474b	2.71	1.98
		qFS16-1	D7	NAU6136	NAU6723a	3.52	-2.12
		qFS16-2	D7	STV023a	TMHM8	3.67	-2.30
		qFS20-1	D10	NAU2139	NAU6463	3.13	2.26
2	FL	FL1	C6	NAU2581			
		FL1	C6	T1			
		FL2	C7	Lc1		10.60	1.56
		FL3	C8	TMB2899		4.00	-0.95
		FL4	C12	N1		4.50	-0.96
	FS	FS1	C6	IT-ISJ07F54R		4.20	0.98
		FS1	C6	NAU2581		10.30	1.27
		FS2	C7	Lc1		13.10	4.13
3	FL	FL1	C6	BNL3650	BNL4108		
		FL2	18/20	BNL3280	AGG/CTC1		
	FS	FS1	3	BNL2530	BNL1434		
		FS2	5	BNL2662	BNL3279		
		FS3	A02/D03	AGC/CAC1	BNL2961		
4	FL	qFL-chr5-1*	5	MUSS193	NAU2865	35.68	7.46
		qFL-chr5-1*	5	MUSS193	NAU2865	33.68	9.45
		qFL-chr5-2*#	5	NAU4034	HAU1316	44.29	2.47
		qFL-chr5-2*#	5	HAU1603	TMB1296	38.92	7.42
		qFL-chr5-2*#	5	NAU4034	HAU1316	54.29	5.32
		qFL-chr5-2*#	5	HAU1315	NAU4034	42.67	7.62
		qFL-chr10-1*#	10	BNL2960	CGR5873	79.66	4.58
		qFL-chr10-1*#	10	CGR5873	GH144	83.43	2.13
		qFL-chr13-1*#	13	DPL687	DPL286	140.49	8.76
		qFL-chr13-1*#	13	DPL687	DPL286	140.49	3.07
		qFL-chr14-1*#	14	HAU1455	GH120	72.60	2.77
		qFL-chr14-1*#	14	CGR6683	HAU1057	83.06	3.15

		qFL-chr14-1*#	14	DPL565	CGR6683	78.05	2.58
		qFL-chr14-2*#	14	HAU1057	NAU3839	83.85	2.47
		qFL-chr14-2*#	14	NAU3308	CGR6802	88.52	3.42
		qFL-chr14-2*#	14	CGR6802	CGR6784	91.93	3.71
		qFL-chr14-2*#	14	BNL3033	BNL2469	84.44	3.25
		qFL-chr3-1	3	CER028	GH663	12.54	3.02
		qFL-chr3-2	3	CGR6528	CGR6017	39.95	3.56
		qFL-chr8-1	8	GH398	DPL090	54.13	3.17
		qFL-chr11-1	11	CGR5602	DPL050a	69.77	4.22
		qFL-chr12-1	12	DPL303	NAU943	13.49	3.77
		qFL-chr13-2	13	BNL1495	DPL687	128.09	3.49
	FS	qFS-chr1-1*	1	DPL182	DC40175	77.62	3.26
		qFS-chr1-1*	1	CIR307	HAU1417b	100.44	3.47
		qFS-chr5-1*#	5	NAU2865	GH388	37.34	3.39
		qFS-chr5-1*#	5	MUSS193	NAU2865	35.68	4.69
		qFS-chr5-1*#	5	NAU2865	GH388	37.34	3.24
		qFS-chr13-1*#	13	DPL687	DPL286	140.49	8.22
		qFS-chr13-1*#	13	DPL687	DPL286	142.49	2.58
		qFS-chr18-1*#	18	CIR099	CIR216	67.40	2.19
		qFS-chr18-1*#	18	DPL077	CIR099	52.20	2.36
		qFS-chr1-2	1	NAU3254	NAU2343	130.31	3.64
		qFS-chr8-1	8	NAU4045	CGR6129	34.52	3.28
		qFS-chr17-1	17	HAU1413a	HAU1417a	0.01	3.61
		qFS-chr18-2	18	BNL243	CER168	19.66	3.29
		qFS-chr24-1	24	NAU4064	CGR6508	99.98	3.03
5	FS	qFS-c9-1	c9	NAU2395b	NAU6101b	5.00	
				NAU1092a	NAU6101b	3.99	
				NAU2395b	NAU6101b	4.12	
				NAU2395	NAU1092a	3.85	
				NAU2395	NAU1092b	3.75	
				NAU2395	NAU1092a	3.80	
				NAU2395	NAU6101	2.32	
				NAU6101	NAU1092	1.85	
6	FL	FL	7	NAU3735	NAU845		
	FS	FS	7	NAU3735	NAU845		
7		qFL-C3-1	C3(A3)	CGR5620		3.89	-0.40
		qFL-C7-1	C7(A7)	DC40182		5.14	-0.54
		qFL-C16-1	C16(D7)	CGR5594		5.97	0.66
			C16(D7)	CGR5139		5.54	0.73
		qFL-C25-1	C25(D6)	HAU2022		8.28	-0.69
		qFS-C2-1	C2(A2)	HAU880		3.86	0.49
		qFS-C7-1	C7(A7)	DC40182		12.52	-1.76
			C7(A7)	SHIN1447		12.34	-1.61

		qFS-C7-2	C7(A7)	DPL0852		11.73	-1.88
8	FL	FL	A4	G1033		16.17	
9	FL	qFL01.1	C1	HAU1044		6.30	
		qFL01.2	C1	NAU3145		4.40	
		qFL03.1	C3	NAU2297		3.10	
		qFL03.2	C3	HAU2065		3.00	
		qFL05.1	C5	HAU3186		4.20	
		qFL08.1	C8	HAU1974		3.10	
		qFL12.1	C12	BNL1605		3.80	
		qFL14.1	C14	MON_DPL0405		3.30	
		qFL15.1	C15	NAU0902		3.50	
		qFL19.1	C19	HAU3213		3.10	
		qFL20.1	C20	NAU3665		3.60	
		qFL23.1	C23	NAU1035		4.00	
		qFL23.2	C23	TMB0515		3.40	
		qFL24.1	C24	Gh397		3.50	
		qFL24.2	C24	NAU1037b		3.40	
		qFLun02.1	Un02	MON_CGR5040		3.50	
	FS	qFS01.1	C1	CIR213		3.00	
		qFS03.1	C3	NAU5233(C3)		3.30	
		qFS07.1	C7	MON_DC40182		6.90	
		qFS23.1	C23	STV022		3.00	
		qFS24.1	C24	MON_CGR5145		5.10	
		qFS24.2	C24	NAU3207		5.10	
		qFS24.3	C24	MON_CGR5870		4.10	
		qFS24.4	C24	Gh268		5.20	
		qFS24.5	C24	MUCS034		5.90	
10	FL	qFL-A5-1	A5	JESPR65	NAU1200	2.82	0.50
		qFL-A5-2	A5	NAU990	NAU1529	2.39	0.43
		qFL-D9-1	D9	BNL1414	BNL1317	2.97	0.46
		qFL-D9-2	D9	BNL1030	JESPR208	4.06	0.50
		qFL-D6-1	D6	JESPR307	TMK12	2.35	–0.40
	FS	qFS-D6-1	D6	BNL4030	NAU1369	3.03	–1.02
		qFS-D6-3	D6	NAU1369	NAU2035	4.13	–0.89
		qFS-D8-1	D8	JESPR127	CIR070a	5.43	–1.20
11	FL	F2-qFL-c1-1	C1	E2M7a		8.74	
		F2-qFL-c1-2	C1	T2E5c		7.60	
		F2-qFL-c6-1	C6	M1E6		3.46	
		F2-qFL-c15-1	C15	BNL1454		2.95	
		F2-qFL-c15-2	C15	BNL2920		2.85	
		F2-qFL-c19-1	C19	CSHE51		3.00	
		F2-qFL-c19-2	C19	CIR364		4.25	
		F2-qFL-c26-1	C26	JESP295a		3.64	

		F2:3-qFL-c3-1	C3	BNL140		2.89	
		F2:3-qFL-c3-2	C3	T3E8b		2.61	
		F2:3-qFL-c6-1	C6	M5E159.3		3.75	
		F2:3-qFL-c9-1	C9	BNL2611b		2.81	
		F2:3-qFL-c9-2	C9	BNL3031b		3.12	
		F2:3-qFL-c9-3	C9	CIR077		2.55	
		F2:3-qFL-c17-1	C17	CSHE109		2.74	
		TC-qFL-c1-1	C1	E2M7a		5.04	
		TC-qFL-c1-2	C1	T2E5c		6.57	
		TC-qFL-c1-3	C1	E4M6d		3.25	
		TC-qFL-c12-1	C12	CIR293		3.09	
		TC-qFL-c12-2	C12	CIR148		4.18	
		TC-qFL-c14-1	C14	BNL3259b		2.64	
		TC-qFL-c26-1	C26	BNL1227a		4.15	
		TC-qFL-c26-2	C26	T5E4		4.16	
	FS	F2-qFS-c4-1	C4	CIR218a		3.50	
		F2-qFS-c7-1	C7	NAU1043		5.13	
		F2-qFS-c14-1	C14	BNL3502		4.15	
		F2-qFS-c14-2	C14	BNL3259b		3.97	
		F2-qFS-c18-1	C18	E2M7f		2.53	
		F2:3-qFS-c8-1	C8	TCE6b		3.03	
		F2:3-qFs-c16-1	C16	CRI100		4.02	
		F2:3-qFS-c16-2	C16	CSHE99b		4.16	
		F2:3-qFS-c23-1	C23	CSHE102a		2.95	
		TC-qFS-c1-1	C1	T2E5c		9.36	
		TC-qFS-c1-2	C1	T4E3a		4.98	
		TC-qFS-c12-1	C12	CIR148		3.85	
		TC-qFS-c23-1	C23	CSHE102a		5.09	
		TC-qFS-c23-2	C23	TM006		4.88	
12	FL	qFL-C4-1	C4	DPL0667		4.60	0.40
		qFL-C4-2	C4	SWU21453		3.40	0.36
		qFL-C4-3	C4	SWU21550		3.50	0.39
		qFL-C4-4	C4	HAU035		3.80	0.65
		qFL-C4-5	C4	SWU16783		3.60	0.49
		qFL-C6-1	C6	MGHES18		3.10	0.47
		qFL-C7-1	C7	PGML00802		4.00	0.49
				NAU2627		4.50	0.59
		qFL-C7-2	C7	NAU1085		2.60	0.44
		qFL-C7-3	C7	NAU3380		3.40	0.36
		qFL-C7-4	C7	HAU244		3.90	0.46
		qFL-C14-1	C14	SWU14535		5.50	-0.53
		qFL-C14-2	C14	SWU14507		5.40	-0.49
		qFL-C14-3	C14	CSHES150		3.20	-0.39

		qFL-C14-4	C14	HAU1455		4.00	-0.48
		qFL-C16-1	C16	SWU10214		3.20	0.42
		qFL-C18-1	C18	CER0168		3.10	-0.42
		qFL-C18-2	C18	NAU2730		3.20	0.43
		qFL-C18-3	C18	NAU5262a		2.23	-0.43
				NAU5262a		2.33	-0.43
				NAU5262a		2.89	-0.55
				NAU5262a		2.11	-0.36
		qFL-C21-1	C21	Gh617		6.78	-0.55
				Gh617		6.61	-0.51
				Gh617		3.90	-0.46
				Gh617		5.77	-0.45
				Gh617		5.93	-0.51
				Gh617		8.60	-0.72
				Gh617		4.02	-0.50
		qFL-C21-2	C21	SWU16050		3.91	-0.50
				SWU16050		3.54	-0.46
		qFL-C21-3	C21	NAU2600		1.28	0.32
		qFL-C22-1	C22	PGML00222		3.50	-0.36
		qFL-C23-1	C23	SWU15058a		13.61	0.56
		qFL-C23-2	C23	DPL0395		2.58	0.45
				DPL0395		3.42	0.55
				DPL0395		2.91	0.56
		qFL-C24-1	C24	NAU6642		4.70	0.39
		qFL-C25-1	C25	SWU19175		2.74	0.42
				Gh537		2.89	0.41
		qFL-C25-2	C25	TMK19		3.02	0.35
				COT012		5.09	0.46
				TMK19		4.84	0.49
				SHIN-1131a		2.82	0.42
		qFL-C25-3	C25	BNL1440		4.32	0.48
				JESPR215		4.68	0.46
				BNL1064		3.38	0.39
				BNL1440		4.53	0.55
				BNL1440		3.37	0.61
				BNL1440		3.06	0.36
		qFL-C25-4	C25	SWU19198		4.92	0.50
				SWU19198		5.86	0.54
				SWU19198		5.90	0.51
				SWU19198		3.41	0.39
				SWU19198		3.25	0.51
		qFL-C25-5	C25	ICRI010391		4.12	0.44
				ICRI010391		3.49	0.43

				DPL0284		4.59	0.47
				ICRI010391		3.50	0.47
				ICRI010391		3.10	0.54
		qFL-C25-6	C25	SWU19320		3.25	0.44
				SWU19320		3.85	0.49
				SWU19320		3.79	0.45
	FS	qFS-C4-1	C4	SWU21453		3.60	0.55
		qFS-C4-2	C4	SWU21550		4.80	0.64
		qFS-C4-3	C4	HAU035		3.30	0.57
		qFS-C6-1	C6	BNL3594b		1.41	-0.36
		qFS-C7-1	C7	PGML00802		11.80	1.11
				PGML00802		9.00	0.82
				PGML00802		7.20	0.92
				PGML00802		9.50	1.10
				PGML00802		9.90	0.95
				PGML00802		4.30	0.91
				PGML00802		6.00	0.98
				PGML00802		12.80	1.24
				NAU2627		5.70	0.84
				PGML00802		6.80	0.75
		qFS-C7-2	C7	NAU1085		4.00	0.68
				NAU1085		3.90	0.65
				NAU1085		4.10	0.74
				NAU1085		5.40	0.66
				NAU1085		4.30	0.80
				NAU1085		4.40	0.74
				NAU1085		4.40	0.60
		qFS-C7-3	C7	NAU3380		3.90	0.59
				NAU3380		4.50	0.64
		qFS-C7-4	C7	C2-0114		3.20	0.60
		qFS-C9-1	C9	SWU15397		4.10	0.82
		qFS-C11-1	C11	SWU16034		1.50	-0.43
		qFS-C12-1	C12	SWU17573b		1.30	-0.40
		qFS-C13-1	C13	SWU21198		1.14	0.32
				SWU21198		1.14	0.32
		qFS-C13-2	C13	MUCS135		3.32	0.50
				MUCS135		3.17	0.60
		qFS-C14-1	C14	SWU14514		2.50	-0.58
		qFS-C14-2	C14	SWU14475		2.00	-0.47
		qFS-C14-3	C14	SWU14535		3.40	-0.55
				SWU14535		3.50	-0.56
				SWU14535		2.20	-0.51
				SWU14535		4.50	-0.80

				SWU14535		4.60	-0.98
				SWU14535		3.20	-0.57
				SWU14535		3.70	-0.84
				SWU14535		5.20	-1.05
		qFS-C14-4	C14	SWU14507		3.10	-0.54
				SWU14507		3.60	-0.51
				SWU14507		4.80	-0.84
				SWU14507		3.40	-0.85
		qFS-C14-5	C14	SWU14506		3.50	-0.77
		qFS-C18-1	C18	PGML00353		1.12	0.32
				NAU6173		3.49	0.66
		qFS-C18-2	C18	SHIN-1452a		1.50	0.38
		qFS-C18-3	C18	TMB2295		1.30	-0.32
		qFS-C19-1	C19	NAU3823		4.41	-0.63
		qFS-C21-1	C21	SWU16370b		3.22	-0.84
		qFS-C21-2	C21	NAU2600		4.59	0.87
		qFS-C23-1	C23	SWU15579		13.85	0.75
		qFS-C23-2	C23	SWU15094		17.13	-0.79
				SWU15094		13.20	-1.13
		qFS-C23-3	C23	SWU15599		12.41	0.99
		qFS-C23-4	C23	PGML00727		12.31	0.72
		qFS-C25-1	C25	DPL0375		2.97	0.78
		qFS-C25-2	C25	Gh537		3.84	0.62
				PGML00463a		4.20	0.86
		qFS-C25-3	C25	TMK19		10.67	0.99
				TMK19		4.04	0.70
				COT012		8.00	0.86
		qFS-C25-4	C25	BNL1440		5.88	1.02
				BNL1440		8.13	0.84
				BNL1440		6.69	0.95
				BNL1440		4.44	0.67
				BNL1440		7.03	1.05
		qFS-C25-5	C25	SWU19198		3.07	0.91
				SWU19198		6.38	1.22
				SWU19198		5.91	0.80
				DC40429		6.12	0.93
				SWU19198		8.08	0.80
				NAU2119		3.33	0.80
		qFS-C25-6	C25	ICRI010391		4.21	0.78
				ICRI010391		3.92	0.87
				DPL0284		5.51	0.83
				DPL0284		6.89	0.80
				DPL0067		5.23	0.84

				ICRI010391		3.30	0.55
				HAU892		5.75	0.95
		qFS-C25-7	C25	SWU19320		3.98	0.75
13	FL	qFL01.1	C1	DPL0490		2.70	0.13
		qFL03.1	C3	BNL2485		2.80	0.67
		qFL05.1	C5	NAU0828		3.00	-0.35
			C5	NAU3212		4.00	0.82
		qFL05.2	C5	NAU2126		3.20	-0.32
		qFL05.3	C5	NAU4951		3.70	-0.20
		qFL06.1	C6	NAU2684		3.40	-0.52
		qFL08.1	C8	BNL3255		3.60	-0.44
		qFL10.1	C10	DPL0600		13.90	-1.40
			C10	CGR5040		4.10	-0.30
		qFL11.1	C11	Gh153		3.60	-0.62
		qFL12.1	C12	JESPR019		3.40	-0.33
		qFL15.1	C15	NAU2165		3.10	-0.43
		qFL18.1	C18	DPL0922		5.00	-0.38
		qFL19.1	C19	DPL0595		2.80	0.46
		qFL19.2	C19	NAU3096		2.80	0.60
		qFL19.3	C19	NAU2708		3.80	1.43
		qFL22.1	C22	DPL0562		2.50	0.40
		qFL23.1	C23	NAU5390		2.90	-0.55
			C23	BNL1414		2.90	-0.35
		qFL23.2	C23	CGR6817		3.20	0.80
		qFL26.1	C26	CGR6012		3.10	0.65
	FS	qFS03.1	C3	BNL2485		2.50	-1.25
		qFS05.1	C5	CGR5732		2.50	-0.51
		qFS06.1	C6	NAU3327		4.50	1.50
		qFS07.1	C7	BNL1694		2.80	0.69
		qFS08.1	C8	NAU3155		4.10	-0.37
		qFS09.1a	C9	HAU0886		2.90	-0.26
		qFS10.1	C10	DPL0207		2.60	-0.15
		qFS10.2	C10	CGR5040		7.70	-2.44
			C10	Gh058		3.20	-0.80
		qFS14.1	C14	JESPR006		3.20	1.75
		qFS15.1	C15	NAU2165		3.30	–1.05
		qFS18.1	C18	Gh060		3.70	0.71
		qFS19.1	C19	DPL0595		3.20	1.07
14	FL	qFL-A7-1	A7	NAU2995			
		qFL-D12-1	D12	NAU4925			
		qFL-D5-1	D5	NAU5489			
		qFL-A11-1	A11	NAU429			
		qFL-A12-1	A12	NAU3109			

		qFL-A12-2	A12	NAU1151			
		qFL-D9-1	D9	NAU3967			
		qFL-D11-1	D11	NAU1366			
		qFL-A5-1	A5	BNL3452			
		qFL-A8-1	A8	NAU3558			
	FS	qFS-A11-2	A11	BNL3431			
		qFS-A9-1	A9	NAU462			
		qFS-A5-2	A5	NAU3014			
		qFS-A11-1	A11	NAU1232			
		qFS-A5-1	A5	BNL3452			
		qFS-D9-1	D9	NAU3967			
		qFS-A8-2	A8	NAU3324			
		qFS-D7-1	D7	NAU3608			
		qFS-D9-2	D9	MUSS151			
		qFS-A7-1	A7	NAU933			
		qFS-D2-1	D2	NAU2987			
		qFS-D1-1	D1	BNL3090			
		qFS-A8-1	A8	NAU3558			
		qFS-A12-1	A12	NAU3109			
		qFS-A12-2	A12	NAU943			
		qFS-D11-1	D11	NAU1366			
15	FL		c21	SHIN-1344	SNP0227	3.36	0.13
			c25	SNP0189	SNP0464	4.08	-0.40
	FS		c06	DPL0080b	SNP0404	2.73	4.27
	FS		c11	DPL0570	DPL1931	4.06	12.85
	FS		c11	DPL0252	DPL1379	3.26	7.52
	FS		c16	SNP0256	SNP0080	2.78	-5.14
	FS		c21	TMB0904	TMB1222b	2.68	-8.31
	FS		c23	JESPR114	DPL1016b	2.97	-4.77
	FS		c25	MUSB0979	DPL0532b	2.89	-5.84
16	FL		C25	SNP0189	SNP0464	4.08	–0.40
	FS		C11	DPL0570	DPL1931	4.06	12.85
17	FS		C24	NAU1295	NAU3499	16.00	10.63
			C24	NAU3071	NAU5399	16.00	11.97
			C24	MUSS280	NAU2631	16.00	11.17
18	FS		Chr03	BNL4034	NAU3541	4.61	0.45
			Chr14	BNL3502	BNL3033	8.18	0.65
			Chr24	CFB5877	GH222	13.44	0.80
			Chr14	CGR6217	CFB5875	8.33	−0.26
			Chr24	CFB5877	GH222	8.94	−0. 2
			Chr14	JESPR006	CFB5875	9.82	0.01
			Chr24	CFB5878	GH222	8.94	0.01
19	FL	qFL-08A-c12-1	c12	NAU3713	BNL0598	4.33	-0.77

		qFL-08A-c21-1	c21	BNL1705	NAU4865	4.29	0.70
		qFL-07W-c11-1	c11	NAU5480	NAU3117	2.65	-0.52
		qFL-07X-c5-1	c5	NAU4057	NAU303	4.34	0.42
	FS	qFS-08A-c21-1	c21	BNL1705	NAU4865	2.72	0.74
		qFS-07A-c20-1	c20	BNL119	NAU3368	3.25	0.43
		qFS-07W-c11-1	c11	NAU5480	NAU3117	3.22	-1.07
		qFS-07X-c11-1	c11	NAU5480	NAU3117	2.70	-0.84
20	FL	FL01.1d	Chr01	A1686a			
		FL01.1d	Chr01	A1686a			
		FL01.1d	Chr01	A1686a			
		FL01.1d	Chr01	A1686a			
		FL01.1d	Chr01	A1686a			
		FL01.1d	Chr01	A1686a			
		FL01.2	Chr01	pGH468			
		FL02.1	Chr02	pGH399			
		FL02.1	Chr02	pGH399			
		FL02.1	Chr02	pGH399			
		FL03.1	Chr03	pAR172b			
		FL03.1d	Chr03	pAR172b			
		FL04.1	Chr04	A1310a			
		FL04.1d	Chr04	A1310a			
		FL05.1	Chr05	pGH530			
		FL05.2	Chr05	G1112a			
		FL05.2	Chr05	G1112a			
		FL06.1	Chr06	A1152			
		FL07.1d	Chr07	G1185a			
		FL07.1	Chr07	G1185a			
		FL12.1d	Chr12	A1252			
		FL12.1d	Chr12	A1252			
		FL14.1d	Chr14	A1727			
		FL14.2d	Chr14	A1580			
		FL15.1	Chr15	pAR077a			
		FL17.1	Chr17	pAR1-56			
		FL17.1	Chr17	pAR1-56			
		FL20.1	Chr20	A1158b			
		FL20.1	Chr20	A1158b			
		FL20.2	Chr20	G1104			
		FL22.1	Chr22	pAR206a			
		FL23.1d	Chr23	pAR547			
		FL23.1d	Chr23	pAR547			
		FL23.1d	Chr23	pAR547			
		FL26.1	Chr26	pAR101b			
		FL26.1	Chr26	pAR101b			

		FL26.1	Chr26	A1310b			
		FLA01.1	LGA01	G1125b			
		FLA02.1	LGA02	pAR792			
		FLA02.1	LGA02	pAR792			
		FLA03.1	LGA03	A1672			
		FLA03.1	LGA03	A1672			
		FLA03.1	LGA03	pAR864			
		FLA03.1	LGA03	pGH243			
		FLA03.1	LGA03	pGH243			
		FLD02.1	LGD02	A1413			
		FLD02.1	LGD02	pAR4-34a			
		FLD02.2	LGD02	A1296			
		FLD03.1	LGD03	pAR571b			
		FLD03.1	LGD03	pAR571b			
		FLD03.1	LGD03	pAR571b			
		FLD03.1	LGD03	pAR571b			
		FLD03.1	LGD03	pAR571b			
		FLD08.1	LGD08	P2-9			
		FLD08.1	LGD08	P2-9			
		FLD08.1	LGD08	P2-9			
		FLD08.2	LGD08	pAR137a			
		FLD08.2	LGD08	pAR137a			
		FLD08.2	LGD08	pAR137a			
		FLD08.2	LGD08	pAR137a			
		FLD08.2	LGD08	pAR137a			
		FLD08.2	LGD08	pAR137a			
21	FL		Chr. 23	BNL1317	NAU701	15.94	0.67
			Chr. 23	BNL1317	TMO06	9.72	0.09
			Chr. 23	BNL1317	TMO06	10.30	0.18
			Chr. 7	NAU474	NAU1043	12.52	-0.15
			Chr. 7	NAU474	NAU1043e	9.63	-0.73
	FS		LGA02	NAU1037	NAU1322	11.03	-0.70
			LGA02	NAU1302	BNL3255	10.38	-0.70
			Chr. 23	NL3140	NAU923	17.97	-0.73
			Chr. 23	NL3383	NAU1004	10.22	-0.99
			Chr. 16	NL1017	BNL1122	12.91	-0.98
			Chr. 16	NL1017	BNL1122	11.27	-0.73
			Chr. 16	NL2634	NAU733	9.93	-0.49
22	FS		C10	OPal19s	OPap01	6.69	–0.91
				OPap01	OPm07	5.23	–1.38
				OPm07	ubc431	6.70	–0.92
				ubc431	SSR2961	7.05	–0.55
				SSR2961	SSR152	6.88	–1.57

				SSR1521	ubc757	6.73	–1.57
				ubc757	ubc301	7.61	–1.65
				OPm07	OPap1	2.71	–1.50
				OPap1	OPal19s	2.78	–0.74
				OPal19s	ubc301	3.41	0.13
				ubc301	SSR2961	3.54	0.17
				OPm07	OPap01	4.94	–1.27
				OPap01	ubc301	5.08	–1.29
				ubc301	OPal19s	4.79	-0.15
				OPal19s	OPap01	1.49	–0.46
				OPap01	OPm07	1.71	–1.04
				OPm07	ubc431	1.49	–0.47
				ubc431	SSR2961	1.53	–0.46
				SSR2961	SSR1521	1.88	–1.10
				SSR1521	ubc757	1.87	–1.10
				ubc757	ubc301	1.98	–1.12
23	FL		Chr09	A1707a			
			Chr20	pAR3			
			LGA01	pAR338a			
			LGA02	pGH530new			
			LGA03	pAR570a			
			LGA05	pAR291a			
	FS		Chr01	A1204			
			Chr01	A1686a			
			Chr04	G1033a			
			Chr14	G1147			
			Chr17	pGH861			
			Chr18	P5			
			Chr18	pAR788			
			Chr20	pGH225			
			Chr22	pAR188			
			Chr22	pAR243			
			Chr23	pAR209			
			Chr25	pGH309			
			LGA01	pAR238			
			LGA02	pGH318b			
			LGA03	pAR570a			
			LGA05	pAR168b			
			LGD02	pAR038			
			LGD03	pAR418a			
			LGD03	pAR503			
			LGD04	pVNC163a			
			LGD07	pGH286			

24	FS	qFSA02a	A02	CIR354	m3e2-420	3.25	–0.62
		qFSA02b	A03	CIR244	BNL3627	3.86	0.16
		qFSchr23	chr23	m8e17-700	m11e11-780	4.58	–1.10
	FL	qFLA02	A02	CIR354	m3e2-420	3.07	–1.49
		qFLchr01	chr01	BNL3580	JESPR289a	6.77	–0.74
		qFLchr06	chr06	m10e10-430	m8e17-300	3.17	–0.43
		qFLchr09	chr09	JESPR230a	BNL1672a	4.54	–0.84
		qFLchr14	chr14	m7e11-360	BNL1059a	3.82	–0.41
25	FL	qFL-LG02-1		NAU4024	NAU3393	4.68	0.33
		qFL-A10-1*		NAU3260	NAU1595	3.33	0.25
				NAU3260	NAU1595	3.44	0.34
		qFL-A10-2		E17M8	NAU1233	2.13	0.24
				E17M8	NAU1233	2.18	0.23
		qFL-D2-1*		CIR246	CIR381b	6.45	–0.41
				CIR246	CIR381b	9.12	–0.46
				CIR246	CIR381b	5.57	–0.41
				CIR246	CIR381b	5.11	–0.35
	FS	qFS-LG05-1		NAU1043	NAU3654	2.30	0.48
		qFS-D2-1		CIR246	CIR381b	2.75	–0.56
				CIR246	CIR381b	2.03	–0.33
		qFS-A11-1		BNL1231	E19M5	2.33	–0.41
		qFS-D9-1*		BNL3140	NAU923	3.86	0.55
26	FL	qFL-D2 - 1	D2	NAU3308		5.75	
		qFL-A11-1	A11	NAU3260		2.42	
		qFL-D2-2	D2	CIR246		3.08	
		qFL-D4-1	D4	NAU5083		2.95	
		qFL-D9-1	D9	BNL1317		3.16	
		qFL-D13-1	D13	BNL3558		4.12	
	FS	qFS-A10-1	A10	NAU2532		5.28	
		qFS-D2 - 1	D2	NAU3308		8.12	
		qFS-A11-1	A11	NAU3260		2.64	
		qFS-D11-1	D11	NAU5091		2.58	
		qFS-D13-1	D13	NAU3948		3.91	
27	FS		LG5	CIR328	BNL3029	5.96	
			9	BNL3031	BNL3410	3.99	
			12	CIR081	CIR148	6.70	
			16	JESPR7	JESPR297	3.63	
			20	BNL3948	BNL2570	2.73	
			26	BNL3482	CIR078	10.05	
	FL		12	CIR081	CIR148	3.54	
			13	JESPR211	CM029	6.11	
			14	CIR381	BNL3034	16.29	
			20	CIR105	CIR238	7.86	

28	FS	qSTR-c1	Chr.01	BNL2599a			
		qSTR-c10	Chr.10	CM0027a			
		qSTR-c11-1	Chr.11	GH329a			
		qSTR-c11-2	Chr.11	CGR6218a			
		qSTR-c13	Chr.13	BNL1421a			
		qSTR-c15-1	Chr.15	TMB0323a			
		qSTR-c15-2	Chr.15	DPL0615a			
		qSTR-c15-2	NA	CIR195a			
		qSTR-c16	Chr.16	CM0066a			
		qSTR-c20	Chr.20	CM0160b			
		qSTR-c21	Chr.21	JESPR238a			
		qSTR-c22	Chr.22	HAU0086b			
		qSTR-c24	Chr.24	BNL1521a			
		qSTR-c26-1	Chr.26	DPL0183a			
		qSTR-c26-1	Chr.26	DPL0028b			
		qSTR-c26-2	Chr.26	DPL0481a			
		qSTR-c4-1	Chr.04	BNL1167a			
		qSTR-c4-2	Chr.04	NAU2477b			
		qSTR-c5-1	Chr.05	TMC005a			
		qSTR-c5-2	Chr.05	SHIN-1552a			
		qSTR-c7-1	Chr.07	C2-0114a			
		qSTR-c7-1	Chr.07	DPL0852a			
		qSTR-c7-1	NA	SHIN-1138b			
		qSTR-c7-1	NA	NAU3822a			
		qSTR-c7-2	Chr.07	SHIN-0438a			
		undetermined	NA	DPL0236a			
	FL	qUHM-c11	Chr.11	SHIN-0966a			
		qUHM-c12	Chr.12	MUSB0846a			
		qUHM-c14	Chr.14	NAU0895a			
		qUHM-c14	NA	HAU3233a			
		qUHM-c16	Chr.16	CM0066a			
		qUHM-c18-1	Chr.18	TMB1208b			
		qUHM-c18-2	Chr.18	DOW006a			
		qUHM-c19-1	Chr.19	DPL0071a			
		qUHM-c19-2	Chr.19	NAU2560a			
		qUHM-c19-2	Chr.19	NAU2217a			
		qUHM-c2-1	Chr.02	BNL1434a			
		qUHM-c2-2	Chr.02	NAU2265a			
		qUHM-c22-1	Chr.22	SHIN-1547a			
		qUHM-c22-1	Chr.22	NAU5046a			
		qUHM-c22-2	Chr.22	NAU5099a			
		qUHM-c22-3	Chr.22	HAU0086b			
		qUHM-c22-3	Chr.22	MUSS145b			

		qUHM-c22-3	Chr.22	HAU0087b			
		qUHM-c22-3	Chr.22	NAU2162a			
		qUHM-c22-3	Chr.22	UCD120b			
		qUHM-c22-3	NA	NAU0913b			
		qUHM-c23	Chr.23	CGR6205a			
		qUHM-c26	Chr.26	DPL0183a			
		qUHM-c26	Chr.26	DPL0028b			
		qUHM-c26	Chr.26	BNL0840a			
		qUHM-c4	Chr.04	NAU2477b			
		qUHM-c4	Chr.04	UCD216b			
		qUHM-c4	Chr.04	NAU2291b			
		qUHM-c5-1	Chr.05	SHIN-1552a			
		qUHM-c5-1	Chr.05	DC40122a			
		qUHM-c5-2	Chr.05	MUSS106a			
		qUHM-c5-2	Chr.05	BNL3400a			
		qUHM-c7	Chr.07	C2-0114a			
		qUHM-c7	Chr.07	DPL0852a			
		qUHM-c7	Chr.07	BNL2733a			
		qUHM-c7	Chr.07	BNL1026a			
		qUHM-c9	Chr.09	MUSS298b			
		qUHM-c9	Chr.09	SHIN-1542a			
		undetermined	NA	SHIN-0272a			
		undetermined	NA	CGR6902b			
		undetermined	NA	DPL1201a			
29	FS	qFS-F2-JES-1a	Chro.D8	TMD05	BNL3145		
		qFS-F2:3-BES-1a	Chro.D8	TMD05	NAU3207		
		qFS-F2-JES-2a	Chro.D8	NAU2665	NAU3605		
		qFS-F2:3-JES-2a	Chro.D8	NAU1534	NAU3605		
		qFS-F2:3-BES-2a	Chro.D8	NAU1534	NAU3499		
		qFS-F2:3-JES-3a	Chro.D8	NAU4099	NAU1534		
		qFS-F2:3-BES-3a	Chro.D8	BNL1521	NAU1534		
		qFS-F2-JES-1b	Chro.D8	NAU1197	NAU780		
		qFS-F2:3-BES-1<br>b	Chro.D8	NAU3201b	TMD05		
		qFS-F2-JES-2b<br>qFS-2 7.15–11.01	Chro.D8	BNL2961	NAU1534		
		qFS-F2:3-BES-2<br>b	Chro.D8	BNL2961	NAU1295		
		qFS-F -BES-3b	Chro.D8	NAU1037	BNL1521		
		qFS-F2-JES-1c	Chro.D8	NAU5379	BNL3474		
		qFS-F2:3-JES-1c	Chro.D8	NAU5379	NAU2926		
		qFS-F2:3-BES-1c	Chro.D8	NAU5379	TMD05		
		qFS-F2-JES-2c	Chro.D8	BNL3474	NAU1322		

		qFS-F2-JES-3c	Chro.D8	NAU3988	NAU3954		
		qFS-F -BES-3c	Chro.D8	NAU3769	NAU5335		
30	FL	FL	A2(Chr.2)	NAU2277			
			A5(Chr.5)	NAU3273			
			A7(Chr.7)	NAU1048			
			A11(Chr.11)	NAU980			
			D3(Chr.17)	NAU3639			
			D4(Chr.22)	JESPR220			
			D10(Chr.20)	NAU3917			
	FS	FS	A10(Chr.10)	cgr5565			
			A11(Chr.11)	NAU980			
			A11(Chr.11)	NAU3390			
			D5(Chr.19)	cgr5510			
31	FL		A2	NAU2277			
			A3	NAU1167			
			A5	NAU1200			
			A5	NAU934			
			A6	NAU2156			
			A8	NAU1037			
			A9	NAU2354			
			A9	BNL1317			
			A10	NAU2508			
			A12	NAU445			
			A12	JESPR295			
			A13	JESPR153			
			D2	NAU2272			
			D5	NAU1102			
			D5	JESPR218			
			D7	BNL2634			
			D7	BNL1395			
			D8	NAU780			
			D8	NAU1302			
			D8	BNL1521			
			D8	JESPR78			
			D8	NAU1336			
			D8	TMD05			
			D8	NAU1262			
			D8	BNL3145			
			D8	NAU1322			
			D8	JESPR127			
			D9	TMO06			
			D10	NAU904			
			D13	NAU2443			

	FS		A5	N U934			
			A7	NAU1043			
			A7	NAU474			
			A8	NAU1037			
			A9	BNL1317			
			A10	NAU2508			
			A12	JESPR295			
			A13	JESPR153			
			D2	NAU2272			
			D6	BNL3436			
			D6	NAU905			
			D7	BNL2634			
			D7	BNL1395			
			D7	BNL1122			
			D8	NAU780			
			D8	NAU816			
			D8	BNL1521			
			D8	JESPR78			
			D8	NAU1197			
			D8	NAU1336			
			D8	TMD05			
			D8	NAU1262			
			D8	BNL3145			
			D8	NAU1322			
			D8	JESPR127			
			D9	TMO06			
			D13	NAU2443			
32	FS			CIR381			
				NAU3419			
	FS			NAU2836			
				BNL3790			
	FS			BNL3347			
	FS			BNL34 6			
	FS			NAU3084			
	FS			HAU1355			
	FS			BNL3436			
	FS			NAU2564			
	FS			NAU6627			
	FL			NAU5480			
	FL			NAU2671			
	FL			NAU6468			
	FL			NAU3092			
	FL			MON_CGR5167			

	FS			STV106			
33	FL		D7	BNL1395			
			UL	DC40182			
			D13	NAU2980			
			D6	NAU2641			
			D10	NAU2776			
			D8	NAU3455			
			D12	NAU3881			
			A4	BNL2572			
			D6	BNL3594			
			D1	CIR30			
			A9	NAU2723			
			D5	NAU3110			
			D13	JESPR153			
			D1	NAU3736			
			A12	NAU3778			
			A1	NAU5411			
			D6	BNL3594			
			D6	BNL827			
			D1	CIR307			
			UL	DC40182			
			5	NAU2894			
			D5	NAU3110			
			A3	NAU3995			
			UL	TMB1618			
	FS			BNL1122			
				BNL2986			
				BNL3408			
				CIR249			
				JESPR6			
34	FL			BNL409			
				BNL542			
				BNL569			
				BNL2495			
				BNL2986			
				BNL3071			
				BNL3090			
				BNL3408			
				BNL3410			
				BNL4017			
				CIR165			
				CIR167			
				CIR170			

				CIR19			

References

1. Xinlei Yang, Xiaodong Zhou, Xingfen Wang, Zhikun Li, YanZhang, Hengwei Liu, Liqiang Wu, Guiyin Zhang, Guijun Yan, Zhiying Ma. Mapping QTL for cotton fiber quality traits using simple sequence repeat markers, conserved intron-scanning primers, and transcript-derived fragments. Euphytica (2015) 201:215–230.
2. Zhengsheng Zhang, Meichun Hu, Jian Zhang, Dajun Liu, Jing Zheng, Ke Zhang, Wei Wang, Qun Wan. Construction of a comprehensive PCR-based marker linkage map and QTL mapping for fiber quality traits in upland cotton (*Gossypium hirsutum* L.). Mol Breeding (2009) 24:49–61.
3. Zhengsheng Zhang, Yuehua Xiao, Ming Luo, Xianbi Li, Xiaoying Luo, Lei Hou, Demou Li & Yan Pei. Construction of a genetic linkage map and QTL analysis of fiber-related traits in upland cotton (*Gossypium hirsutum* L.). Euphytica (2005) 144: 91–99.
4. Qingzhi Liang, Cheng Hu, Hua Hua, ZhaoHu Li & Jinping Hua. Construction of a linkage map and QTL mapping for fiber quality traits in upland cotton (*Gossypium hirsutum* L.). Chinese Science Bulletin Plant Genetics. (2013) 58: 3233-3243.
5. X. Yang, Y. Wang, G. Zhang, X. Wang, L. Wu, H. Ke, H. Liu, Z. Ma. Detection and validation of one stable fiber strength QTL on c9 in tetraploid cotton. Mol Genet Genomics (2016) 291:1625–1638.
6. Zhibin Cao, Xiefei Zhu, Hong Chen, Tianzhen Zhang. Fine mapping of clustered quantitative trait loci for fiber quality on chromosome 7 using a *Gossypium barbadense* introgressed line. Mol Breeding (2015) 35:215
7. Furong Wang, Zhenzhen Xu, Ran Sun, Yongchao Gong, Guodong Liu, Jingxia Zhang, Liuming Wang, Chuanyun Zhang, Shoujin Fan, Jun Zhang. Genetic dissection of the introgressive genomic components from *Gossypium barbadense* L. that contribute to improved fiber quality in *Gossypium hirsutum* L. Mol Breeding (2013) 32:547–562.
8. M. Mei, N. H. Syed, W. Gao, P. M. Thaxton, C. W. Smith, D. M. Stelly, Z. J. Chen. Genetic mapping and QTL analysis of fiber-related traits in cotton (*Gossypium*). Theor Appl Genet (2004) 108:280–291
9. Ke Zhang, Jian Zhang, Jing Ma, Shiyi Tang, Dajun Liu, Zhonghua Teng, Dexin Liu, Zhengsheng Zhang. Genetic mapping and quantitative trait locus analysis of fiber quality traits using a three-parent composite population in upland cotton (*Gossypium hirsutum* L.). Mol Breeding (2012) 29:335–348.
10. Xinlian Shen, Wangzhen Guo, Qiongxian Lu, Xiefei Zhu, Youlu Yuan, Tianzhen Zhang. Genetic mapping of quantitative trait loci for fiber quality and yield trait by RIL approach in upland cotton. Euphytica (2007) 155:371–380.
11. Jiwen Yu, Shuxun Yu, Michael Gore, Man Wu, Honghong Zhai, Xingli Li, Shuli Fan, Meizhen Song, Jinfa Zhang. Identification of quantitative trait loci across interspecific F2, F2:3 and testcross populations for agronomic and fiber traits in tetraploid cotton. Euphytica (2013) 191:375–389.
12. Muhammad Jamshed, Fei Jia, Juwu Gong, Koffi Kibalou Palanga, Yuzhen Shi, Junwen Li, Haihong Shang, Aiying Liu, Tingting Chen, Zhen Zhang, Juan Cai, Qun Ge, Zhi Liu, Quanwei Lu, Xiaoying Deng, Yunna Tan, Harun or Rashid, Zareen Sarfraz, Murtaza Hassan, Wankui Gong and Youlu Yuan. Identification of stable quantitative trait loci (QTLs) for fiber quality traits across multiple environments in *Gossypium hirsutum* recombinant inbred line population. BMC Genomics (2016) 17:197.
13. Qianshun Shao, Fengjiao Zhang, Shiyi Tang, Yong Liu, Xiaomei Fan, Dexin Liu, Dajun Liu, Jian Zhang, Zhonghua Teng, Andrew H. Paterson, Zhengsheng Zhang. Identifying QTL for fiber quality traits with three upland cotton (*Gossypium hirsutum* L.) populations. Euphytica (2014) 198:43–58.

14. Peng Wang, Yajuan Zhu, Xianliang Song, Zhibin Cao, Yezhang Ding, Bingliang Liu, Xiefei Zhu, Sen Wang, Wangzhen Guo, Tianzhen Zhang. Inheritance of long staple fiber quality traits of *Gossypiumbarbadense* in *G. hirsutum* background using CSILs. Theor Appl Genet (2012) 124:1415–1428.
15. Michael A. Gore, David D. Fang, Jesse A. Poland, Jinfa Zhang, Richard G. Percy, Roy G. Cantrell, Gregory Thyssen and Alexander E. Lipka. Linkage map construction and QTL analysis of agronomic and fiber quality traits in cotton. The Plant Genome (2013)22: 1-62.
16. Michael A. Gore, David D. Fang, Jesse A. Poland, Jinfa Zhang, Richard G. Percy, Roy G. Cantrell, Gregory Thyssen and Alexander E. Lipka. Linkage map construction and quantitative trait locus analysis of agronomic and fiber quality traits in cotton. The plant genome (2014)7: 1-10.
17. Pawan Kumar, Rippy Singh, Edward L. Lubbers, Xinlian Shen, Andrew H. Paterson, B. Todd Campbell, Donald C. Jones, and Peng W. Chee. Mapping and validation of fiber strength quantitative trait loci on chromosome 24 in upland cotton. Crop Science (2012) 52: 1115-1122.
18. Md. S. Islam, Linghe Zeng, Gregory N. Thyssen, Christopher D. Delhom, Hee Jin Kim, Ping Li, David D. Fang. Mapping by sequencing in cotton (*Gossypium hirsutum*) line MD52ne identified candidate genes for fiber strength and its related quality attributes. Theor Appl Genet (2016) 129:1071–1086
19. Jiwen Yu, Ke Zhang, Shuaiyang Li, Shuxun Yu, Honghong Zhai, Man Wu, Xingli Li, Shuli Fan, Meizhen Song, Daigang Yang, Yunhai Li, Jinfa Zhang. Mapping quantitative trait loci for lint yield and fiber quality across environments in a *Gossypium hirsutum* × *Gossypium barbadense* backcross inbred line population. Theor Appl Genet (2013) 126:275–287.
20. Peng W. Chee, Xavier Draye, Chunxiao Jiang, Laura Decanini, Terrie A. Delmonte Robert Bredhauer, C. Wayne Smith, Andrew H. Paterson. Molecular dissection of phenotypic variation between *Gossypium hirsutum* and *Gossypium barbadense* (cotton) by a backcross-self approach: III. Fiber length. Theor Appl Genet (2005) 111: 772–781.
21. Xinlian Shen, Wangzhen Guo, Xiefei Zhu, Youlu Yuan, John Z. Yu, Russell J. Kohel and Tianzhen Zhang. Molecular mapping of QTLs for fiber qualities in three diverse lines in upland cotton using SSR markers. Molecular Breeding (2005) 15: 169–181.
22. Tianzhen Zhang, Youlu Yuan, John Yu, Wangzhen Guo, Russell J. Kohel. Molecular tagging of a major QTL for fiber strength in Upland cotton and its marker-assisted selection. Theor Appl Genet (2003) 106:262–268
23. A. H. Paterson, Y. Saranga, M. Menz, C. X. Jiang, R. J. Wright. QTL analysis of genotype × environment interactions affecting cotton fiber quality. Theor Appl Genet (2003) 106:384–396.
24. Daohua He, Zhongxu Lin, Xianlong Zhang, Yichun Nie,Xiaoping Guo, Yanxin Zhang, Wu Li. QTL mapping for economic traits based on a dense genetic map of cotton with PCR-based markers using the interspecific cross of *Gossypium hirsutum* × *Gossypium barbadense*. Euphytica (2007) 153:181–197.
25. Baohua Wang, Wangzhen Guo, Xiefei Zhu, Yaoting Wu, Naitai Huang, Tianzhen Zhang. QTL mapping of fiber quality in an elite hybrid derived-RIL population of upland cotton. Euphytica (2006) 152:367–378.
26. Hongde Qin, Wangzhen Guo, Yuanming Zhang, Tianzhen Zhang. QTL mapping of yield and fiber traits based on a four-way cross population in *Gossypium hirsutum* L. Theor Appl Genet (2008) 117:883–894.
27. Jixiang Wu, Osman Ariel Gutierrez, Johnie N. Jenkins, Jack C. McCarty, Jun Zhu. Quantitative analysis and QTL mapping for agronomic and fiber traits in an RI population of upland cotton. Euphytica (2009) 165:231–245.
28. David D. Fang, Johnie N. Jenkins, Dewayne D. Deng, Jack C. McCarty, Ping Li and Jixiang Wu. Quantitative trait loci analysis of fiber quality traits using a random-mated recombinant inbred population in upland cotton (*Gossypium hirsutum* L.). BMC Genomics 2014, 15:397.
29. Hong Chen, Neng Qian, Wangzhen Guo, Qingping Song, Baocheng Li, Fujun Deng, Cunguang Dong,

Tianzhen Zhang. Using three overlapped RILs to dissect genetically clustered QTL for Wber strength on Chro.D8 in upland cotton. Theor Appl Genet (2009) 119:605–612.

30. Tianzhen Zhang, Neng Qian, Xiefei Zhu, HongChen, Sen Wang, Hongxian Mei, Yuanming Zhang. Variations and transmission of QTL alleles for yield and fiber qualities in upland cotton cultivars developed in China. PLoS ONE (2013) 8(2): e57220.

31. Caiping Cai, Wenxue Ye, Tianzhen Zhang and Wangzhen Guo. Association analysis of fiber quality traits andexploration of elite alleles in upland cottoncultivars/accessions (*Gossypium hirsutum* L.). Journal of Integrative Plant Biology (2014) 56:151–162.

32. Xinhui Nie, Cong Huang, Chunyuan You, Wu Li, Wenxia Zhao, Chao Shen, Beibei Zhang, Hantao Wang, Zhenhua Yan, Baoshen Dai, Maojun Wang, Xianlong Zhang and Zhongxu Lin. Genome-wide SSR-based association mapping for fiber quality in nation-wide upland cotton inbreed cultivars in China. BMC Genomics (2016) 17:352.

33. Hongde Qin, Min Chen, Xianda Yi, Shu Bie, Cheng Zhang, Youchang Zhang, Jiayang Lan, Yanyan Meng, Youlu Yuan, Chunhai Jiao. Identification of associated ssr markers for yield component and fiber quality traits based on frame map and upland cotton collections. PLoS ONE (2015) 10(1): e0118073.

34. Linghe Zeng, William R. Meredith Jr, Osman A. Gutie ́rrez, Deborah L. Boykin. Identification of associations between SSR markers and fiber traits in an exotic germplasm derived from multiple crosses among *Gossypium* tetraploid species. Theor Appl Genet (2009) 119:93–103

Table S8 SNP markers near physical locations of 268 SSR markers and their corresponding p value associated with FL

SSR primers	Chro.	L-position	R-position	*p*	SNP loci	-lg(p)
NAU474	Dt7	26072040	26072348	0.000898	rsDt7:26072147	3.05
GH663	Dt3	19364941	19365031	0.001966	rsDt3:19398516	2.71
BNL3145	Dt5	38884420	38884660	0.004432	rsDt5:38888704	2.35
JESPR215	Dt10	16653657	16653787	0.006991	rsDt10:16667181	2.16
NAU4024	At3	41444876	41445069	0.009813	rsAt3:41489628	2.01
NBRI_Gh_B008	At1	69402820	69403062	0.010411	rsAt1:69474725	1.98
NAU934	At11	37343222	37343427	0.017488	rsAt11:37342756	1.76
BNL119	Dt11	57666111	57666331	0.020196	rsDt11:57665108	1.69
SWU16034	At1	53379349	53379515	0.020293	rsAt1:53107700	1.69
NAU3954	At4	75180764	75181219	0.020627	rsAt4:75184632	1.69
BNL3474	At4	68302854	68303025	0.022153	rsAt4:68290472	1.65
SWU21453	At9	22195383	22195586	0.022925	rsAt9:22194353	1.64
BNL1317	At6	55146322	55146504	0.022926	rsAt6:55202787	1.64
CIR078	At5	8179074	8179420	0.026025	rsAt5:8143385	1.58
BNL1440	Dt10	26542994	26543231	0.029441	rsDt10:26584621	1.53
NAU3014	At9	38127042	38127242	0.030031	rsAt9:38073202	1.52
CIR364	Dt9	55697071	55697223	0.036891	rsDt9:55652578	1.43
NAU1043	Dt1	41015811	41016047	0.038607	rsDt1:40097268	1.41
BNL3502	Dt5	64246171	64246327	0.043625	rsDt5:64268480	1.36
NAU3917	Dt11	260152	260328	0.045802	rsDt11:245193	1.34
CIR216	At4	37892657	37892800	0.053284	rsAt4:37893762	1.27
PGML00727	Dt6	27861462	27861562	0.05513	rsDt6:27824025	1.26
NAU905	At1	14696121	14696281	0.056889	rsAt1:14667149	1.24
JESPR65	At9	65248273	65248412	0.066138	rsAt9:65265835	1.18
BNL2920	Dt2	10488974	10489130	0.071982	rsDt2:10487011	1.14
BNL2961	At5	30079153	30079373	0.07204	rsAt5:30152304	1.14
NAU2987	At5	25152708	25152928	0.07209	rsAt5:24929878	1.14
NAU3954	Dt4	4376161	4376613	0.072558	rsDt4:4367640	1.14
NAU2126	Dt9	56553336	56553522	0.075184	rsDt9:56555898	1.12
Gh058	At4	88750448	88750685	0.075303	rsAt4:88756925	1.12
HAU2065	At5	2164191	2164527	0.077053	rsAt5:2162635	1.11
NAU904	At11	69233103	69233288	0.077433	rsAt11:69246498	1.11
NAU3393	Dt5	49520044	49520236	0.077481	rsDt5:49540927	1.11
CIR099	At8	75394193	75394279	0.08218	rsAt8:75401388	1.09
CIR307	Dt2	10798015	10798185	0.083361	rsDt2:10812646	1.08
NAU2302	At9	89240067	89240229	0.084015	rsAt9:89175340	1.08
BNL1694	Dt1	22853205	22853454	0.087358	rsDt1:22784135	1.06
JESPR295	Dt8	3105722	3105825	0.089438	rsDt8:3113052	1.05
NAU6173	At2	9160675	9160822	0.091624	rsAt2:9159509	1.04
NAU2156	At10	45691132	45691258	0.093215	rsAt10:45699295	1.03
BNL1694	Dt1	61881625	61881848	0.096822	rsDt1:61855186	1.01

SWU16783	At12	52490118	52490282	0.104364	rsAt12:52246649	0.98
Gh537	Dt10	22517518	22517690	0.105978	rsDt10:22384556	0.97
NAU3390	At1	3847161	3847349	0.106797	rsAt1:3854638	0.97
NAU5083	Dt12	1570807	1571183	0.106797	rsDt12:1595728	0.97
BNL3033	At5	21337425	21337573	0.107183	rsAt5:21600989	0.97
NAU980	Dt9	42307522	42307688	0.107633	rsDt9:42407316	0.97
NAU5091	At1	11299871	11300044	0.108479	rsAt1:11280577	0.96
BNL1122	Dt1	79102465	79102628	0.11075	rsDt1:79349996	0.96
SWU14507	Dt5	25358636	25358824	0.119195	rsDt5:25351165	0.92
BNL1705	Dt7	7832088	7832278	0.123095	rsDt7:7926156	0.91
JESPR220	Dt12	3354573	3354733	0.124352	rsDt12:3350389	0.91
HAU3186	Dt11	4514368	4514567	0.131549	rsDt11:4410204	0.88
NAU2684	At10	32279370	32279530	0.136053	rsAt10:32278403	0.87
HAU3213	Dt9	64016145	64016357	0.136202	rsDt9:64016034	0.87
NAU3541	At5	46782784	46782924	0.140292	rsAt5:46785294	0.85
SWU10214	Dt12	18725753	18725921	0.141789	rsDt12:18811170	0.85
NAU3096	Dt9	39851268	39851452	0.144448	rsDt9:39828431	0.84
SWU21453	Dt12	8135385	8135578	0.156619	rsDt12:8101639	0.81
NAU3207	Dt4	12370729	12370970	0.164697	rsDt4:12361314	0.78
NAU3735	Dt1	39835154	39835893	0.166456	rsDt1:39825610	0.78
SWU21453	Dt1	53830738	53830941	0.16681	rsDt1:53811407	0.78
JESPR127	At4	12383356	12383576	0.168803	rsAt4:12385482	0.77
BNL3140	Dt6	10639249	10639355	0.168829	rsDt6:10652722	0.77
NAU3014	Dt9	47562455	47562639	0.17528	rsDt9:47638948	0.76
NAU2035	Dt10	8591758	8592001	0.177973	rsDt10:8567504	0.75
BNL1231	At1	2980982	2981180	0.178134	rsAt1:2943775	0.75
CIR364	At3	2384338	2384481	0.181395	rsAt3:2357252	0.74
BNL3033	Dt5	38483084	38483250	0.18308	rsDt5:38353862	0.74
NAU3308	Dt5	38483108	38483332	0.18308	rsDt5:38353862	0.74
HAU1057	Dt5	38483174	38483332	0.18308	rsDt5:38353862	0.74
NAU3541	Dt5	18193380	18193567	0.193103	rsDt5:18196052	0.71
NAU2581	Dt10	44859390	44859633	0.194115	rsDt10:44850184	0.71
NAU5379	Dt4	37165102	37165259	0.202785	rsDt4:37168488	0.69
BNL2570	Dt11	6312387	6312621	0.205094	rsDt11:6307768	0.69
BNL3279	At1	2512654	2512769	0.221858	rsAt1:2525664	0.65
HAU0886	Dt6	39975254	39975471	0.22195	rsDt6:40456505	0.65
NAU972	Dt5	15369981	15370166	0.223006	rsDt5:15380847	0.65
SWU14506	Dt5	25439584	25439754	0.235675	rsDt5:25439670	0.63
NAU3665	Dt11	56754070	56754292	0.24107	rsDt11:56750160	0.62
NAU3036	At10	47317275	47317463	0.247577	rsAt10:47319107	0.61
NAU3393	At3	85892135	85892321	0.24948	rsAt3:85904946	0.60
NAU3948	At13	85922753	85922940	0.25134	rsAt13:85946406	0.60
NAU1366	At7	6618160	6621923	0.254198	rsAt7:6796441	0.59

NAU1366	At7	6618160	6618384	0.254198	rsAt7:6796441	0.59
NAU1366	At7	6621708	6621923	0.254198	rsAt7:6796441	0.59
CIR293	Dt8	49720495	49720797	0.260213	rsDt8:49727441	0.58
NAU1035	At6	3703644	3703815	0.260623	rsAt6:3828671	0.58
NAU4925	At8	42744792	42744931	0.26213	rsAt8:42743694	0.58
NAU3273	At9	59996920	59997111	0.263201	rsAt9:59955506	0.58
NAU5379	Dt4	15285802	15285959	0.266125	rsDt4:15280570	0.57
NAU3212	At9	45318273	45318443	0.269368	rsAt9:45387861	0.57
NAU0828	Dt9	55189774	55189967	0.271348	rsDt9:55241004	0.57
NAU3096	Dt9	42565424	42565599	0.285123	rsDt9:42572196	0.54
NAU2627	Dt1	46597190	46597387	0.285274	rsDt1:46596698	0.54
BNL3580	At2	7799109	7799325	0.288942	rsAt2:7750439	0.54
Gh268	Dt11	41840896	41841011	0.301349	rsDt11:41837612	0.52
JESPR211	Dt7	26462771	26462974	0.302998	rsDt7:26438041	0.52
CM029	Dt7	26462779	26462935	0.302998	rsDt7:26438041	0.52
NAU1322	Dt4	35035586	35035760	0.303597	rsDt4:35009130	0.52
NAU1048	Dt1	39985739	39985949	0.304971	rsDt1:39985713	0.52
NAU972	At5	46385736	46385981	0.309045	rsAt5:46442803	0.51
NAU913	Dt8	2358734	2358930	0.31221	rsDt8:2334435	0.51
BNL3031	Dt6	16496225	16496409	0.318121	rsDt6:16507821	0.50
BNL3280	Dt13	29127635	29127866	0.31936	rsDt13:29123373	0.50
MUSB0979	At10	57284799	57284967	0.320279	rsAt10:57299631	0.49
NAU3608	At9	47396413	47396655	0.322034	rsAt9:47252041	0.49
SWU14475	Dt5	34039771	34039880	0.3263	rsDt5:33998708	0.49
MON_CGR5145	Dt4	28863531	28863665	0.331062	rsDt4:28804448	0.48
Gh060	Dt13	20085660	20085819	0.331101	rsDt13:20061332	0.48
NAU1366	Dt7	3637314	3637538	0.334014	rsDt7:3781748	0.48
GH144	At13	33882749	33882916	0.342906	rsAt13:33882290	0.46
NAU3988	Dt4	17027774	17027993	0.347448	rsDt4:17038354	0.46
NAU5399	Dt13	8225584	8225760	0.353401	rsDt13:8228033	0.45
NAU1035	Dt7	10157755	10157926	0.354699	rsDt7:10190919	0.45
NAU2987	Dt5	32721538	32721758	0.357057	rsDt5:32738206	0.45
NAU2272	Dt5	19364176	19364342	0.358276	rsDt5:19394917	0.45
TMB2899	At1	51959321	51959569	0.359582	rsAt1:51967898	0.44
BNL3029	Dt9	57282236	57282362	0.371468	rsDt9:57231361	0.43
NAU1262	Dt4	21909322	21909551	0.375956	rsDt4:21928867	0.42
NAU6642	Dt4	12962207	12962393	0.378269	rsDt4:12914106	0.42
BNL4034	Dt5	22508220	22508296	0.380024	rsDt5:22463690	0.42
NAU3207	At4	62027593	62027837	0.380459	rsAt4:62024517	0.42
NAU4024	Dt5	50567677	50570579	0.381257	rsDt5:50552982	0.42
NAU4024	Dt5	50567677	50567870	0.381257	rsDt5:50552982	0.42
NAU4024	Dt5	50570389	50570579	0.381257	rsDt5:50552982	0.42
JESPR211	At8	76629641	76629842	0.392048	rsAt8:76911263	0.41

CM029	At8	76629649	76629803	0.392048	rsAt8:76911263	0.41
SWU16050	At1	54855988	54856195	0.39557	rsAt1:54885764	0.40
Gh617	At1	54856025	54856186	0.39557	rsAt1:54885764	0.40
MUSS193	At9	51305085	51305286	0.400143	rsAt9:51296149	0.40
GH144	Dt11	25032055	25032228	0.405207	rsDt11:24964510	0.39
JESPR78	Dt10	7741457	7741786	0.410366	rsDt10:7738105	0.39
JESPR208	Dt6	6744041	6744154	0.422142	rsDt6:6744787	0.37
CIR081	Dt8	49786315	49786540	0.425998	rsDt8:49792837	0.37
SWU16783	Dt8	3105694	3105852	0.437301	rsDt8:3050985	0.36
NAU1043	Dt7	25085387	25085612	0.439727	rsDt7:24999787	0.36
NAU1102	At4	5334525	5334765	0.44093	rsAt4:5283713	0.36
Gh153	Dt13	47820540	47820744	0.442988	rsDt13:47900539	0.35
NAU5390	Dt6	4069107	4069401	0.445484	rsDt6:4062064	0.35
JESPR114	Dt6	3175918	3176012	0.456427	rsDt6:3150819	0.34
NAU3988	At4	7663235	7663437	0.459803	rsAt4:7655574	0.34
BNL3029	At9	44650927	44651055	0.461467	rsAt9:44636215	0.34
JESPR218	Dt9	5719802	5719920	0.467884	rsDt9:5728125	0.33
NAU3713	Dt8	56416770	56416915	0.470436	rsDt8:56446421	0.33
TMB1296	Dt9	66815268	66815492	0.475366	rsDt9:66815733	0.32
HAU1315	Dt9	67179488	67179732	0.482972	rsDt9:67132527	0.32
HAU1316	Dt9	67179713	67180097	0.482972	rsDt9:67132527	0.32
NAU980	At5	1341033	1341199	0.483385	rsAt5:1342691	0.32
NAU4951	Dt9	22332244	22332540	0.486886	rsDt9:22353938	0.31
NAU2165	At1	24312200	24312404	0.492575	rsAt1:24195553	0.31
NAU2165	At1	24319895	24320099	0.492575	rsAt1:24195553	0.31
GH663	At5	14175423	14175527	0.498414	rsAt5:14266565	0.30
NAU2302	At4	77040737	77040927	0.504965	rsAt4:76842667	0.30
NAU1151	At10	64940705	64940914	0.514413	rsAt10:64912779	0.29
NAU0828	Dt11	3259270	3259469	0.529688	rsDt11:3236551	0.28
JESPR211	Dt11	38657425	38657628	0.544625	rsDt11:38656966	0.26
CM029	Dt11	38657433	38657589	0.544625	rsDt11:38656966	0.26
BNL3948	Dt11	20574670	20574770	0.546912	rsDt11:20629593	0.26
NAU1085	At9	77264383	77264622	0.556106	rsAt9:77284805	0.25
NAU3967	Dt6	12699048	12699288	0.559549	rsDt6:12639293	0.25
NAU3254	Dt9	51349026	51349318	0.563611	rsDt9:51348204	0.25
NAU2343	Dt9	51349026	51349288	0.563611	rsDt9:51348204	0.25
NAU3499	Dt10	6778957	6779182	0.565596	rsDt10:6787594	0.25
NAU2926	Dt4	21782345	21782566	0.569818	rsDt4:21763910	0.24
NAU2277	At4	23362406	23362545	0.573332	rsAt4:23364746	0.24
GH222	Dt10	11277366	11277522	0.576279	rsDt10:11240446	0.24
NAU5489	Dt9	50067192	50067433	0.580689	rsDt9:50069545	0.24
BNL3145	Dt5	38884420	38884660	0.58166	rsDt5:39004929	0.24
BNL3255	Dt1	96136004	96139773	0.581921	rsDt1:96195198	0.24

NAU1369	At10	76565796	76566037	0.587921	rsAt10:76571286	0.23
BNL3034	Dt5	18614359	18614515	0.593402	rsDt5:18618497	0.23
SWU10214	Dt1	44941526	44941713	0.594648	rsDt1:44969425	0.23
BNL1231	At1	4179712	4179910	0.59955	rsAt1:4378377	0.22
TMB0515	At6	54496343	54496534	0.606785	rsAt6:54519403	0.22
BNL1440	At10	79780504	79780763	0.616556	rsAt10:79760598	0.21
NAU2119	At10	79782959	79783199	0.616556	rsAt10:79760598	0.21
SWU19320	At10	79912353	79912530	0.616556	rsAt10:79760598	0.21
NAU3769	Dt4	28458399	28458642	0.622744	rsDt4:28445621	0.21
BNL3090	At2	14818257	14818497	0.629024	rsAt2:14743078	0.20
HAU0886	Dt6	34043184	34043401	0.634677	rsDt6:34044937	0.20
SWU14507	At5	38520823	38520994	0.635657	rsAt5:38560037	0.20
SWU14507	At5	38555386	38555557	0.635657	rsAt5:38560037	0.20
BNL1317	Dt6	8194386	8194602	0.651019	rsDt6:8235471	0.19
SWU15397	Dt6	9084897	9085113	0.657191	rsDt6:9076621	0.18
SWU16050	Dt7	18060524	18060740	0.660309	rsDt7:17943573	0.18
HAU1455	At5	40470802	40471055	0.664666	rsAt5:40465896	0.18
NAU923	At6	53465863	53466091	0.668438	rsAt6:53455197	0.17
BNL3452	Dt9	65608970	65609160	0.669696	rsDt9:65557737	0.17
HAU3186	At9	57891448	57891647	0.669968	rsAt9:57862926	0.17
GH388	Dt9	67110621	67110766	0.670182	rsDt9:67132247	0.17
NAU4034	Dt9	67110690	67110869	0.670182	rsDt9:67132247	0.17
HAU1057	At5	21337343	21337483	0.677147	rsAt5:21137027	0.17
NAU2165	Dt2	28752503	28752687	0.681537	rsDt2:28755006	0.17
BNL1122	At7	16828982	16829155	0.688152	rsAt7:16750793	0.16
HAU1455	Dt5	21966091	21966349	0.694806	rsDt5:21942786	0.16
NAU2865	Dt9	66807687	66808043	0.695056	rsDt9:66814040	0.16
NAU1068	At9	73495670	73495925	0.703385	rsAt9:73508698	0.15
NAU2995	Dt1	58408983	58409180	0.715152	rsDt1:58225123	0.15
PGML00802	Dt1	80162098	80162386	0.73871	rsDt1:80125201	0.13
NAU4057	At9	57115051	57115255	0.742838	rsAt9:57085243	0.13
NAU1295	Dt13	8922830	8922991	0.746282	rsDt13:8923448	0.13
NAU3558	At1	52085828	52086030	0.747794	rsAt1:52091180	0.13
BNL3410	Dt6	13896003	13896224	0.749161	rsDt6:13975368	0.13
NAU3368	Dt11	46979114	46979356	0.751922	rsDt11:46871947	0.12
NAU1068	At12	7172426	7172681	0.754697	rsAt12:7134522	0.12
NAU990	Dt7	11116495	11116702	0.755147	rsDt7:11113549	0.12
TMB0904	Dt7	31575847	31576048	0.757457	rsDt7:31353462	0.12
BNL2960	At13	617563	617712	0.759549	rsAt13:642976	0.12
NAU2708	Dt9	58569377	58572300	0.759602	rsDt9:58578292	0.12
NAU2708	Dt9	58569377	58569604	0.759602	rsDt9:58578292	0.12
NAU2708	Dt9	58572087	58572300	0.759602	rsDt9:58578292	0.12
HAU1974	At4	13192789	13192937	0.761871	rsAt4:13184431	0.12

NAU1322	At4	13192798	13192996	0.761871	rsAt4:13184431	0.12
NAU3665	At13	8433555	8433746	0.765387	rsAt13:8452711	0.12
NAU4034	At9	49419673	49419853	0.772588	rsAt9:49416846	0.11
HAU1316	At9	49902524	49902914	0.813611	rsAt9:49917794	0.09
BNL1317	Dt6	8194386	8194602	0.822821	rsDt6:8171646	0.08
NAU2730	At13	90345774	90345979	0.823082	rsAt13:90343979	0.08
SWU19175	Dt10	20029849	20030059	0.827451	rsDt10:20013015	0.08
NAU3464	Dt5	9456931	9457231	0.828205	rsDt5:9458069	0.08
SWU19198	Dt10	21721751	21721861	0.829512	rsDt10:21718150	0.08
BNL1495	At13	85882340	85882455	0.830804	rsAt13:85880597	0.08
NAU3654	Dt1	41746071	41746273	0.832671	rsDt1:41734700	0.08
NAU3212	Dt9	57898190	57898348	0.833546	rsDt9:57678485	0.08
NAU1295	At5	29798534	29798695	0.835683	rsAt5:29799156	0.08
JESPR211	At13	20671521	20671734	0.837593	rsAt13:20642363	0.08
CM029	At13	20671560	20671726	0.837593	rsAt13:20642363	0.08
SWU15094	Dt6	21777369	21777515	0.838691	rsDt6:21674732	0.08
NAU6136	Dt8	28246856	28247148	0.840373	rsDt8:28043872	0.08
SWU19175	At10	70862537	70862733	0.848586	rsAt10:70982483	0.07
NAU5083	At12	51816646	51817025	0.852138	rsAt12:51933359	0.07
MON_CGR5870	Dt13	9512760	9512896	0.864847	rsDt13:9526355	0.06
BNL4034	At5	39544147	39544232	0.865768	rsAt5:39561087	0.06
BNL3482	Dt8	45027300	45027438	0.874415	rsDt8:45027556	0.06
NAU2684	Dt10	1529137	1529291	0.891752	rsDt10:1493091	0.05
HAU035	At12	50842003	50842193	0.892416	rsAt12:50854287	0.05
CIR246	At1	105935980	105936146	0.895209	rsAt1:105923025	0.05
HAU035	Dt6	8504670	8504862	0.901925	rsDt6:8546789	0.04
NAU3273	At9	62577990	62579623	0.903006	rsAt9:62561496	0.04
NAU3273	At9	62577990	62578184	0.903006	rsAt9:62561496	0.04
NAU3273	At9	62579429	62579623	0.903006	rsAt9:62561496	0.04
NAU2995	Dt1	24240481	24240657	0.909014	rsDt1:24160787	0.04
JESPR153	At13	9256503	9256631	0.91271	rsAt13:9263440	0.04
NAU1262	At4	89977999	89978228	0.915845	rsAt4:89944249	0.04
NAU905	At10	37459995	37460161	0.933135	rsAt10:37354150	0.03
BNL3558	Dt13	52897171	52897380	0.935667	rsDt13:52899236	0.03
NAU2631	Dt4	35032885	35033071	0.938282	rsDt4:35027489	0.03
HAU1974	Dt4	35035577	35035701	0.938282	rsDt4:35027489	0.03
NAU1322	Dt4	35035586	35035760	0.938282	rsDt4:35027489	0.03
BNL1064	At10	13490050	13490194	0.938847	rsAt10:13462185	0.03
NAU3071	Dt10	8895644	8895848	0.949295	rsDt10:8897026	0.02
Gh397	Dt13	8081218	8081295	0.959172	rsDt13:8131341	0.02
BNL1454	Dt2	9147666	9147768	0.959503	rsDt2:9154138	0.02
NAU2926	At4	83604752	83604952	0.964892	rsAt4:83763232	0.02
GH120	Dt5	22814938	22815107	0.965337	rsDt5:22812743	0.02

CIR078	Dt8	51633132	51633479	0.982139	rsDt8:51619583	0.01
CIR213	At9	85706390	85706624	0.988894	rsAt9:85701386	0.00
SWU14514	Dt5	23462135	23462235	0.999166	rsDt5:23432556	0.00

Table S9 SNP markers near physical locations of 268 SSR markers and their corresponding *p* value associated with FS

SSR primers	Chro	L-position	R-position	p	SNP loci	-lg(p)
NAU3954	At4	75180764	75181219	0.00088	rsAt4:75184632	3.06
SWU21453	At9	22195383	22195586	0.001701	rsAt9:22194353	2.77
NAU3014	At9	38127042	38127242	0.004868	rsAt9:38073202	2.31
BNL3474	At4	68302854	68303025	0.005905	rsAt4:68290472	2.23
BNL2920	Dt2	10488974	10489130	0.006916	rsDt2:10487011	2.16
NAU1322	Dt4	35035586	35035760	0.00802	rsDt4:35009130	2.10
BNL3145	Dt5	38884420	38884660	0.01466	rsDt5:38888704	1.83
JESPR215	Dt10	16653657	16653787	0.020229	rsDt10:16667181	1.69
CIR216	At4	37892657	37892800	0.020821	rsAt4:37893762	1.68
NAU4024	At3	41444876	41445069	0.021254	rsAt3:41489628	1.67
HAU3213	Dt9	64016145	64016357	0.027029	rsDt9:64016034	1.57
GH663	Dt3	19364941	19365031	0.032858	rsDt3:19398516	1.48
SWU16783	At12	52490118	52490282	0.034721	rsAt12:52246649	1.46
CIR078	At5	8179074	8179420	0.036008	rsAt5:8143385	1.44
NAU6173	At2	9160675	9160822	0.038062	rsAt2:9159509	1.42
BNL1694	Dt1	22853205	22853454	0.038862	rsDt1:22784135	1.41
NAU2156	At10	45691132	45691258	0.039579	rsAt10:45699295	1.40
NAU923	At6	53465863	53466091	0.03986	rsAt6:53455197	1.40
JESPR65	At9	65248273	65248412	0.039898	rsAt9:65265835	1.40
NAU980	Dt9	42307522	42307688	0.040528	rsDt9:42407316	1.39
NAU905	At1	14696121	14696281	0.047902	rsAt1:14667149	1.32
SWU14507	Dt5	25358636	25358824	0.061995	rsDt5:25351165	1.21
BNL119	Dt11	57666111	57666331	0.066108	rsDt11:57665108	1.18
BNL3140	Dt6	10639249	10639355	0.066363	rsDt6:10652722	1.18
NAU3390	At1	3847161	3847349	0.079326	rsAt1:3854638	1.10
NAU2684	At10	32279370	32279530	0.085425	rsAt10:32278403	1.07
NAU2987	At5	25152708	25152928	0.092042	rsAt5:24929878	1.04
BNL3034	Dt5	18614359	18614515	0.093403	rsDt5:18618497	1.03
BNL4034	Dt5	22508220	22508296	0.09351	rsDt5:22463690	1.03
BNL3502	Dt5	64246171	64246327	0.093923	rsDt5:64268480	1.03
NAU2035	Dt10	8591758	8592001	0.099891	rsDt10:8567504	1.00
NAU5379	Dt4	37165102	37165259	0.103929	rsDt4:37168488	0.98
JESPR127	At4	12383356	12383576	0.112474	rsAt4:12385482	0.95
PGML00727	Dt6	27861462	27861562	0.124414	rsDt6:27824025	0.91
NAU934	At11	37343222	37343427	0.12554	rsAt11:37342756	0.90
NAU1043	Dt1	41015811	41016047	0.128681	rsDt1:40097268	0.89
NAU2302	At9	89240067	89240229	0.130178	rsAt9:89175340	0.89
NAU1295	Dt13	8922830	8922991	0.139537	rsDt13:8923448	0.86
CIR099	At8	75394193	75394279	0.141372	rsAt8:75401388	0.85
NAU2987	Dt5	32721538	32721758	0.142252	rsDt5:32738206	0.85
NAU904	At11	69233103	69233288	0.14419	rsAt11:69246498	0.84

SWU16034	At1	53379349	53379515	0.145885	rsAt1:53107700	0.84
SWU14506	Dt5	25439584	25439754	0.150758	rsDt5:25439670	0.82
NAU0828	Dt9	55189774	55189967	0.152891	rsDt9:55241004	0.82
NAU5083	Dt12	1570807	1571183	0.153893	rsDt12:1595728	0.81
Gh537	Dt10	22517518	22517690	0.15747	rsDt10:22384556	0.80
BNL3280	Dt13	29127635	29127866	0.16339	rsDt13:29123373	0.79
SWU16783	Dt8	3105694	3105852	0.17045	rsDt8:3050985	0.77
NAU1366	Dt7	3637314	3637538	0.173968	rsDt7:3781748	0.76
JESPR295	Dt8	3105722	3105825	0.175369	rsDt8:3113052	0.76
NAU3014	Dt9	47562455	47562639	0.182318	rsDt9:47638948	0.74
JESPR211	At8	76629641	76629842	0.184538	rsAt8:76911263	0.73
CM029	At8	76629649	76629803	0.184538	rsAt8:76911263	0.73
NAU3735	Dt1	39835154	39835893	0.186205	rsDt1:39825610	0.73
BNL1440	Dt10	26542994	26543231	0.193712	rsDt10:26584621	0.71
MON_CGR5145	Dt4	28863531	28863665	0.198037	rsDt4:28804448	0.70
NAU913	Dt8	2358734	2358930	0.20396	rsDt8:2334435	0.69
NAU1035	At6	3703644	3703815	0.208929	rsAt6:3828671	0.68
HAU2065	At5	2164191	2164527	0.216768	rsAt5:2162635	0.66
NAU3096	Dt9	39851268	39851452	0.219717	rsDt9:39828431	0.66
CIR307	Dt2	10798015	10798185	0.221612	rsDt2:10812646	0.65
NAU2126	Dt9	56553336	56553522	0.224134	rsDt9:56555898	0.65
NAU3254	Dt9	51349026	51349318	0.224334	rsDt9:51348204	0.65
NAU2343	Dt9	51349026	51349288	0.224334	rsDt9:51348204	0.65
HAU1455	Dt5	21966091	21966349	0.231515	rsDt5:21942786	0.64
NAU3917	Dt11	260152	260328	0.234486	rsDt11:245193	0.63
BNL2961	At5	30079153	30079373	0.236618	rsAt5:30152304	0.63
BNL1122	Dt1	79102465	79102628	0.240186	rsDt1:79349996	0.62
BNL3033	Dt5	38483084	38483250	0.240573	rsDt5:38353862	0.62
NAU3308	Dt5	38483108	38483332	0.240573	rsDt5:38353862	0.62
HAU1057	Dt5	38483174	38483332	0.240573	rsDt5:38353862	0.62
CIR293	Dt8	49720495	49720797	0.241041	rsDt8:49727441	0.62
NAU3207	Dt4	12370729	12370970	0.241284	rsDt4:12361314	0.62
BNL1317	At6	55146322	55146504	0.248581	rsAt6:55202787	0.60
BNL3580	At2	7799109	7799325	0.250637	rsAt2:7750439	0.60
NAU3096	Dt9	42565424	42565599	0.254793	rsDt9:42572196	0.59
CIR364	Dt9	55697071	55697223	0.266147	rsDt9:55652578	0.57
NAU3954	Dt4	4376161	4376613	0.271347	rsDt4:4367640	0.57
HAU3186	At9	57891448	57891647	0.27439	rsAt9:57862926	0.56
NAU3988	Dt4	17027774	17027993	0.27948	rsDt4:17038354	0.55
HAU1315	Dt9	67179488	67179732	0.283919	rsDt9:67132527	0.55
HAU1316	Dt9	67179713	67180097	0.283919	rsDt9:67132527	0.55
NAU3558	At1	52085828	52086030	0.285051	rsAt1:52091180	0.55
BNL2570	Dt11	6312387	6312621	0.288579	rsDt11:6307768	0.54

TMB0904	Dt7	31575847	31576048	0.290648	rsDt7:31353462	0.54
NAU4024	Dt5	50567677	50570579	0.301073	rsDt5:50552982	0.52
NAU4024	Dt5	50567677	50567870	0.301073	rsDt5:50552982	0.52
NAU4024	Dt5	50570389	50570579	0.301073	rsDt5:50552982	0.52
NAU2581	Dt10	44859390	44859633	0.304514	rsDt10:44850184	0.52
NAU1048	Dt1	39985739	39985949	0.321257	rsDt1:39985713	0.49
Gh058	At4	88750448	88750685	0.323004	rsAt4:88756925	0.49
GH144	Dt11	25032055	25032228	0.326854	rsDt11:24964510	0.49
BNL3031	Dt6	16496225	16496409	0.328442	rsDt6:16507821	0.48
Gh268	Dt11	41840896	41841011	0.330885	rsDt11:41837612	0.48
BNL3029	Dt9	57282236	57282362	0.33237	rsDt9:57231361	0.48
NAU6136	Dt8	28246856	28247148	0.349406	rsDt8:28043872	0.46
CIR213	At9	85706390	85706624	0.350217	rsAt9:85701386	0.46
NAU3541	At5	46782784	46782924	0.351421	rsAt5:46785294	0.45
NAU1262	Dt4	21909322	21909551	0.351919	rsDt4:21928867	0.45
JESPR211	Dt7	26462771	26462974	0.365769	rsDt7:26438041	0.44
CM029	Dt7	26462779	26462935	0.365769	rsDt7:26438041	0.44
NAU2730	At13	90345774	90345979	0.369327	rsAt13:90343979	0.43
NAU1102	At4	5334525	5334765	0.370195	rsAt4:5283713	0.43
HAU1057	At5	21337343	21337483	0.373875	rsAt5:21137027	0.43
NAU3541	Dt5	18193380	18193567	0.381428	rsDt5:18196052	0.42
NAU1262	At4	89977999	89978228	0.382025	rsAt4:89944249	0.42
NAU1366	At7	6618160	6621923	0.385006	rsAt7:6796441	0.41
NAU1366	At7	6618160	6618384	0.385006	rsAt7:6796441	0.41
NAU1366	At7	6621708	6621923	0.385006	rsAt7:6796441	0.41
NAU3713	Dt8	56416770	56416915	0.38827	rsDt8:56446421	0.41
NAU5399	Dt13	8225584	8225760	0.391716	rsDt13:8228033	0.41
NAU6642	Dt4	12962207	12962393	0.395435	rsDt4:12914106	0.40
NAU2627	Dt1	46597190	46597387	0.401286	rsDt1:46596698	0.40
BNL3033	At5	21337425	21337573	0.403711	rsAt5:21600989	0.39
PGML00802	Dt1	80162098	80162386	0.408624	rsDt1:80125201	0.39
NAU3393	At3	85892135	85892321	0.41172	rsAt3:85904946	0.39
NAU1043	Dt7	25085387	25085612	0.416183	rsDt7:24999787	0.38
BNL3255	Dt1	96136004	96139773	0.425634	rsDt1:96195198	0.37
NBRI_Gh_B008	At1	69402820	69403062	0.434003	rsAt1:69474725	0.36
GH663	At5	14175423	14175527	0.440568	rsAt5:14266565	0.36
BNL3948	Dt11	20574670	20574770	0.440641	rsDt11:20629593	0.36
NAU2302	At4	77040737	77040927	0.442183	rsAt4:76842667	0.35
NAU3988	At4	7663235	7663437	0.446349	rsAt4:7655574	0.35
NAU1369	At10	76565796	76566037	0.454264	rsAt10:76571286	0.34
NAU3212	At9	45318273	45318443	0.456752	rsAt9:45387861	0.34
TMB1296	Dt9	66815268	66815492	0.458324	rsDt9:66815733	0.34
BNL1694	Dt1	61881625	61881848	0.460766	rsDt1:61855186	0.34

NAU5083	At12	51816646	51817025	0.468473	rsAt12:51933359	0.33
BNL3279	At1	2512654	2512769	0.469039	rsAt1:2525664	0.33
GH144	At13	33882749	33882916	0.474467	rsAt13:33882290	0.32
NAU980	At5	1341033	1341199	0.479844	rsAt5:1342691	0.32
GH120	Dt5	22814938	22815107	0.481686	rsDt5:22812743	0.32
CIR078	Dt8	51633132	51633479	0.48172	rsDt8:51619583	0.32
Gh153	Dt13	47820540	47820744	0.4826	rsDt13:47900539	0.32
NAU3393	Dt5	49520044	49520236	0.482664	rsDt5:49540927	0.32
MUSB0979	At10	57284799	57284967	0.485016	rsAt10:57299631	0.31
BNL1440	At10	79780504	79780763	0.485123	rsAt10:79760598	0.31
NAU2119	At10	79782959	79783199	0.485123	rsAt10:79760598	0.31
SWU19320	At10	79912353	79912530	0.485123	rsAt10:79760598	0.31
JESPR218	Dt9	5719802	5719920	0.485864	rsDt9:5728125	0.31
HAU0886	Dt6	39975254	39975471	0.487811	rsDt6:40456505	0.31
NAU1085	At9	77264383	77264622	0.493477	rsAt9:77284805	0.31
JESPR211	At13	20671521	20671734	0.501359	rsAt13:20642363	0.30
CM029	At13	20671560	20671726	0.501359	rsAt13:20642363	0.30
JESPR114	Dt6	3175918	3176012	0.501618	rsDt6:3150819	0.30
SWU10214	Dt1	44941526	44941713	0.504084	rsDt1:44969425	0.30
NAU4925	At8	42744792	42744931	0.511478	rsAt8:42743694	0.29
NAU2995	Dt1	58408983	58409180	0.513092	rsDt1:58225123	0.29
NAU2708	Dt9	58569377	58572300	0.515104	rsDt9:58578292	0.29
NAU2708	Dt9	58569377	58569604	0.515104	rsDt9:58578292	0.29
NAU2708	Dt9	58572087	58572300	0.515104	rsDt9:58578292	0.29
SWU10214	Dt12	18725753	18725921	0.516921	rsDt12:18811170	0.29
TMB2899	At1	51959321	51959569	0.519314	rsAt1:51967898	0.28
NAU3036	At10	47317275	47317463	0.528613	rsAt10:47319107	0.28
NAU3499	Dt10	6778957	6779182	0.538983	rsDt10:6787594	0.27
BNL1231	At1	2980982	2981180	0.541163	rsAt1:2943775	0.27
HAU3186	Dt11	4514368	4514567	0.541522	rsDt11:4410204	0.27
SWU15397	Dt6	9084897	9085113	0.542527	rsDt6:9076621	0.27
NAU3948	At13	85922753	85922940	0.54633	rsAt13:85946406	0.26
JESPR78	Dt10	7741457	7741786	0.549824	rsDt10:7738105	0.26
NAU990	Dt7	11116495	11116702	0.5543	rsDt7:11113549	0.26
NAU4951	Dt9	22332244	22332540	0.560139	rsDt9:22353938	0.25
BNL3145	Dt5	38884420	38884660	0.565533	rsDt5:39004929	0.25
MON_CGR5870	Dt13	9512760	9512896	0.570129	rsDt13:9526355	0.24
NAU1295	At5	29798534	29798695	0.575302	rsAt5:29799156	0.24
HAU1316	At9	49902524	49902914	0.577659	rsAt9:49917794	0.24
NAU3608	At9	47396413	47396655	0.580831	rsAt9:47252041	0.24
BNL3558	Dt13	52897171	52897380	0.58139	rsDt13:52899236	0.24
SWU14507	At5	38520823	38520994	0.591952	rsAt5:38560037	0.23
SWU14507	At5	38555386	38555557	0.591952	rsAt5:38560037	0.23

HAU1455	At5	40470802	40471055	0.601072	rsAt5:40465896	0.22
SWU21453	Dt12	8135385	8135578	0.604753	rsDt12:8101639	0.22
NAU1068	At12	7172426	7172681	0.604962	rsAt12:7134522	0.22
NAU5489	Dt9	50067192	50067433	0.608555	rsDt9:50069545	0.22
NAU5379	Dt4	15285802	15285959	0.613161	rsDt4:15280570	0.21
TMB0515	At6	54496343	54496534	0.613695	rsAt6:54519403	0.21
NAU3769	Dt4	28458399	28458642	0.614883	rsDt4:28445621	0.21
NAU3665	Dt11	56754070	56754292	0.622181	rsDt11:56750160	0.21
BNL2960	At13	617563	617712	0.633696	rsAt13:642976	0.20
NAU3207	At4	62027593	62027837	0.634205	rsAt4:62024517	0.20
NAU3967	Dt6	12699048	12699288	0.642784	rsDt6:12639293	0.19
MUSS193	At9	51305085	51305286	0.645928	rsAt9:51296149	0.19
BNL1317	Dt6	8194386	8194602	0.651877	rsDt6:8235471	0.19
BNL1705	Dt7	7832088	7832278	0.652203	rsDt7:7926156	0.19
HAU035	Dt6	8504670	8504862	0.655963	rsDt6:8546789	0.18
SWU16050	Dt7	18060524	18060740	0.657311	rsDt7:17943573	0.18
JESPR208	Dt6	6744041	6744154	0.657571	rsDt6:6744787	0.18
NAU3212	Dt9	57898190	57898348	0.666899	rsDt9:57678485	0.18
BNL3090	At2	14818257	14818497	0.676885	rsAt2:14743078	0.17
BNL4034	At5	39544147	39544232	0.677489	rsAt5:39561087	0.17
SWU21453	Dt1	53830738	53830941	0.678119	rsDt1:53811407	0.17
BNL1454	Dt2	9147666	9147768	0.679521	rsDt2:9154138	0.17
NAU2926	Dt4	21782345	21782566	0.68743	rsDt4:21763910	0.16
JESPR220	Dt12	3354573	3354733	0.694696	rsDt12:3350389	0.16
GH388	Dt9	67110621	67110766	0.699312	rsDt9:67132247	0.16
NAU4034	Dt9	67110690	67110869	0.699312	rsDt9:67132247	0.16
SWU14475	Dt5	34039771	34039880	0.699333	rsDt5:33998708	0.16
NAU1151	At10	64940705	64940914	0.701139	rsAt10:64912779	0.15
NAU1035	Dt7	10157755	10157926	0.705638	rsDt7:10190919	0.15
NAU2272	Dt5	19364176	19364342	0.706371	rsDt5:19394917	0.15
NAU4034	At9	49419673	49419853	0.723428	rsAt9:49416846	0.14
NAU3654	Dt1	41746071	41746273	0.724443	rsDt1:41734700	0.14
NAU3368	Dt11	46979114	46979356	0.726773	rsDt11:46871947	0.14
GH222	Dt10	11277366	11277522	0.727614	rsDt10:11240446	0.14
BNL3410	Dt6	13896003	13896224	0.727689	rsDt6:13975368	0.14
NAU1068	At9	73495670	73495925	0.740835	rsAt9:73508698	0.13
NAU2165	At1	24312200	24312404	0.742345	rsAt1:24195553	0.13
NAU2165	At1	24319895	24320099	0.742345	rsAt1:24195553	0.13
NAU905	At10	37459995	37460161	0.742855	rsAt10:37354150	0.13
NAU3071	Dt10	8895644	8895848	0.743745	rsDt10:8897026	0.13
SWU15094	Dt6	21777369	21777515	0.745479	rsDt6:21674732	0.13
Gh397	Dt13	8081218	8081295	0.746206	rsDt13:8131341	0.13
BNL1317	Dt6	8194386	8194602	0.748045	rsDt6:8171646	0.13

HAU035	At12	50842003	50842193	0.750399	rsAt12:50854287	0.12
NAU3273	At9	59996920	59997111	0.751974	rsAt9:59955506	0.12
SWU19198	Dt10	21721751	21721861	0.768563	rsDt10:21718150	0.11
BNL1231	At1	4179712	4179910	0.772364	rsAt1:4378377	0.11
BNL3482	Dt8	45027300	45027438	0.779876	rsDt8:45027556	0.11
HAU0886	Dt6	34043184	34043401	0.789408	rsDt6:34044937	0.10
CIR246	At1	1.06E+08	1.06E+08	0.790131	rsAt1:105923025	0.10
NAU972	At5	46385736	46385981	0.791926	rsAt5:46442803	0.10
CIR364	At3	2384338	2384481	0.807143	rsAt3:2357252	0.09
CIR081	Dt8	49786315	49786540	0.807579	rsDt8:49792837	0.09
BNL3029	At9	44650927	44651055	0.827927	rsAt9:44636215	0.08
NAU2277	At4	23362406	23362545	0.832717	rsAt4:23364746	0.08
BNL1064	At10	13490050	13490194	0.838963	rsAt10:13462185	0.08
JESPR153	At13	9256503	9256631	0.847553	rsAt13:9263440	0.07
NAU3464	Dt5	9456931	9457231	0.851381	rsDt5:9458069	0.07
Gh060	Dt13	20085660	20085819	0.858134	rsDt13:20061332	0.07
NAU2865	Dt9	66807687	66808043	0.862045	rsDt9:66814040	0.06
BNL1495	At13	85882340	85882455	0.87425	rsAt13:85880597	0.06
BNL1122	At7	16828982	16829155	0.875903	rsAt7:16750793	0.06
NAU2926	At4	83604752	83604952	0.882925	rsAt4:83763232	0.05
NAU4057	At9	57115051	57115255	0.889183	rsAt9:57085243	0.05
BNL3452	Dt9	65608970	65609160	0.894817	rsDt9:65557737	0.05
NAU2165	Dt2	28752503	28752687	0.89875	rsDt2:28755006	0.05
SWU16050	At1	54855988	54856195	0.899164	rsAt1:54885764	0.05
Gh617	At1	54856025	54856186	0.899164	rsAt1:54885764	0.05
NAU2684	Dt10	1529137	1529291	0.903131	rsDt10:1493091	0.04
NAU0828	Dt11	3259270	3259469	0.90466	rsDt11:3236551	0.04
NAU972	Dt5	15369981	15370166	0.912038	rsDt5:15380847	0.04
NAU2995	Dt1	24240481	24240657	0.915363	rsDt1:24160787	0.04
SWU19175	Dt10	20029849	20030059	0.920693	rsDt10:20013015	0.04
JESPR211	Dt11	38657425	38657628	0.927078	rsDt11:38656966	0.03
CM029	Dt11	38657433	38657589	0.927078	rsDt11:38656966	0.03
HAU1974	At4	13192789	13192937	0.933593	rsAt4:13184431	0.03
NAU1322	At4	13192798	13192996	0.933593	rsAt4:13184431	0.03
SWU19175	At10	70862537	70862733	0.938004	rsAt10:70982483	0.03
NAU3273	At9	62577990	62579623	0.938903	rsAt9:62561496	0.03
NAU3273	At9	62577990	62578184	0.938903	rsAt9:62561496	0.03
NAU3273	At9	62579429	62579623	0.938903	rsAt9:62561496	0.03
NAU5390	Dt6	4069107	4069401	0.938925	rsDt6:4062064	0.03
NAU5091	At1	11299871	11300044	0.960178	rsAt1:11280577	0.02
NAU2631	Dt4	35032885	35033071	0.991853	rsDt4:35027489	0.00
HAU1974	Dt4	35035577	35035701	0.991853	rsDt4:35027489	0.00
NAU1322	Dt4	35035586	35035760	0.991853	rsDt4:35027489	0.00

SWU14514	Dt5	23462135	23462235	0.992191	rsDt5:23432556	0.00
NAU3665	At13	8433555	8433746	0.992252	rsAt13:8452711	0.00

Table S10. Information on 355 upland cotton germplasms.

Serial number	Variety names	Geographic origin	Serial number	Variety names	Geographic origin	Serial number	Variety names	Geographic origin
1	zhong02191	HY	51	liaomian7	LN	101	zhongmiansuo64	HY
2	zhong040418	HY	52	liaomian9	LN	102	zhongmiansuo74	HY
3	zhong040618	HY	53	liaoyangduanjie	LN	103	776	HY
4	zhong040712	HY	54	lu154	HY	104	20398	HY
5	zhong051811	HY	55	lu890	HY	105	61930	HY
6	zhong051822	HY	56	lumina2153	HY	106	61995	HY
7	zhong061832	HY	57	nongken5	NW	107	102909	HY
8	zhong071239	HY	58	shan70	HY	108	103026	HY
9	PB12-1-10	HY	59	shizao1	HY	109	103028	HY
10	PB12-1-7	HY	60	shizao2	HY	110	103030	HY
11	PB12-1-8	HY	61	shizao3	HY	111	103032	HY
12	zhong1476	HY	62	xia13-7	HY	112	103075	HY
13	zhong151222	HY	63	xia25	HY	113	103164	HY
14	zhong152201	HY	64	xiazao1	HY	114	103245	HY
15	SQ152201	HY	65	xiazao2	HY	115	103297	HY
16	zhong152214	HY	66	xiazao3	HY	116	109056	HY
17	SQ152224	HY	67	xinluzao11	NW	117	SPPB12-1-7	HY
18	2011SS	HY	68	xinluzao36	NW	118	SPPB12-1-9	HY
19	QS2012-3	HY	69	xinluzao3	NW	119	SPPB12-2-7	HY
20	QS2012-4	HY	70	xinluzao42	NW	120	298	HY
21	29-41	HY	71	xinluzao45	NW	121	602186	HY
22	29-42	HY	72	xinluzao4	NW	122	CG3020-1	USA
23	6426	HY	73	xinluzao6	NW	123	CG3020-3	USA
24	K640	HZY	74	xinluzao8	NW	124	FM1735	USA
25	N82	HZY	75	xinluzao9	NW	125	G2005	HY

26	P21-6-7	HZY	76	xinxiang368	HY	126	GK44	HY
27	SF06	HY	77	yu1335	HY	127	H109	HY
28	SGK16	HZY	78	yumian12	HY	128	H559	HY
29	V321-20-14	HZY	79	yuzao8E13	HY	129	LIH33	HY
30	baimian17	HY	80	yuzao910	HY	130	Phy-7	USA
31	chaoyangmian1	LN	81	yunzaoN177	HY	131	STS458	USA
32	Deltapine20	USA	82	yunzaoN95	HY	132	TM-1	USA
33	Delfos97-047	USA	83	zhong416	HY	133	aoshimian4406	HY
34	guannong1	LN	84	zhong425-5	HY	134	baimian985	HY
35	han2490	HY	85	zhong716	HY	135	cang198	HY
36	han656	HY	86	zhong751213	HY	136	chunbeibao	HY
37	han559	HY	87	zhongchuang88	HY	137	chunnanbao	HY
38	han667	HY	88	zhongmiansuo10	HY	138	Deltapine14	USA
39	han686	HY	89	zhongmiansuo14	HY	139	Deltapine15	USA
40	han9609	HY	90	zhongmiansuo16	HY	140	fanmian3	HY
41	heishanmian1	LN	91	zhongmiansuo20	HY	141	ganzao109	HZY
42	jinmian3	LN	92	zhongmiansuo24	HY	142	guoxinmian11	HY
43	jinmian10	HY	93	zhongmiansuo27	HY	143	han7860	HY
44	jinmian21	HY	94	zhongmiansuo30	HY	144	ji4025	HY
45	jinmian23	HY	95	zhongmiansuo36	HY	145	jinmian26	HY
46	jinmian5	HY	96	han256	HY	146	BM03	HY
47	liaomian10	LN	97	zhongmiansuo37	HY	147	kelin098	HY
48	liaomian17	LN	98	zhongmiansuo42	HY	148	liaomian23	LN
49	liaomian5	LN	99	zhongmiansuo50	HY	149	liaomian27	LN
50	liaomian6	LN	100	zhongmiansuo58	HY	150	liaomian28	LN

151	lu05R59	HY	201	chuan01	HZY	251	xinluzhong6	NW
152	lu7619	HY	202	xinluzao2	NW	252	xinluzhong7	NW
153	lumianyan17	HY	203	xinluzao10	NW	253	xinluzhong8	NW
154	lumianyan21	HY	204	xinluzao12	NW	254	xinluzhong9	NW
155	lumianyan28	HY	205	xinluzao13	NW	255	xinluzhong10	NW
156	lumianyan36	HY	206	xinluzao15	NW	256	xinluzhong12	NW
157	lumianyan38	HY	207	xinluzao16	NW	257	xinluzhong13	NW
158	miaobao21	HY	208	xinluzao17	NW	258	xinluzhong14	NW
159	renhe39	HY	209	xinluzao18	NW	259	xinluzhong15	NW
160	rihuimian6	HZY	210	xinluzao19	NW	260	xinluzhong16	NW
161	shannongSF01	HY	211	xinluzao20	NW	261	xinluzhong17	NW
162	shan79	HY	212	xinluzao21	NW	262	xinluzhong19	NW
163	Stoneville2B	USA	213	xinluzao22	NW	263	xinluzhong20	NW
164	xinmian33B	USA	214	xinluzao23	NW	264	xinluzhong21	NW
165	xinzhimian5	HY	215	xinluzao24	NW	265	xinluzhong22	NW
166	yinhuashu	HY	216	yumian5	HY	266	xinluzhong26	NW
167	you009	HY	217	yumian18	HY	267	xinluzhong27	NW
168	zhong109	HY	218	yumian21	HY	268	xinluzhong28	NW
169	zhongmiansuo17	HY	219	yun1729	HY	269	xinluzhong30	NW
170	zhongmiansuo19	HY	220	zhemian11	HZY	270	xinluzhong32	NW
171	zhongmiansuo43	HY	221	Tkuo	NW	271	xinluzhong34	NW
172	zhongmiansuo60	HY	222	bo425	NW	272	xinluzhong35	NW
173	zhong662	HY	223	xinluzhong60	NW	273	xinluzhong40	NW
174	zhong679	HY	224	kenN27-3	NW	274	xinluzhong41	NW
175	zhongmiansuo69	HY	225	B-3	NW	275	xinluzhong45	NW

176	zhong800319	HY	226	xinluzao25	NW	276	xinluzhong46	NW
177	zhong915	HY	227	xinluzao26	NW	277	xinluzhong47	NW
178	zhongmiansuo12	HY	228	xinluzao27	NW	278	KZGL	USA
179	zhongmiansuo35	HY	229	xinluzao28	NW	279	jiangyin1	HZY
180	zhongmiansuo41	HY	230	xinluzao29	NW	280	huihe36	NW
181	zhongmiansuo45	HY	231	xinluzao30	NW	281	xinluzao60	NW
182	zhongmiansuo49	HY	232	xinluzao32	NW	282	guoxinmian9	HY
183	zhongmiansuo7	HY	233	xinluzao33	NW	283	junmian1	NW
184	zhongzhimian8	HY	234	xinluzao34	NW	284	huiyuan717	NW
185	zhongzhimianGD89	HY	235	xinluzao35	NW	285	yunzao219	HY
186	shan920346	HY	236	xinluzao37	NW	286	yunzao33-356	HY
187	xianIII9704	NW	237	xinluzao38	NW	287	jinmian2	LN
188	USA-1	USA	238	xinluzao39	NW	288	chaoyangmian2	LN
189	JEJS	Others	239	xinluzao40	NW	289	dunmian77-116	NW
190	bazhou5409	NW	240	xinluzao41	NW	290	ganmian4	NW
191	yumian1	HZY	241	xinluzao46	NW	291	guannongzaoC-50	LN
192	huazhong910102	HZY	242	xinluzao47	NW	292	gunagnongchangzao14	LN
193	yiselie	Others	243	xinluzao48	NW	293	yanzao1	HZY
194	ken6614	NW	244	xinluzao49	NW	294	yanzao2	HZY
195	ken0074	NW	245	xinluzao50	NW	295	liaojinmian6	LN
196	chuan239-1	HZY	246	xinluzao51	NW	296	jinken69-2	NW
197	bamian3 号	NW	247	xinluzhong1	NW	297	jinken148-39	NW
198	chuan338	HZY	248	xinluzhong3	NW	298	zhuangjiahan102	NW
199	chaun267	HZY	249	xinluzhong4	NW	299	yinmian4	HY
200	chuan65	HZY	250	xinluzhong5	NW	300	ejing1	HZY

301	simian3	HZY	326	xiangmian13	HZY	351	xuzhou219	HZY
302	Stoneville4B	USA	327	yapengmian	HZY	352	yinmian8	HY
303	jimian10	HY	328	jijiaodezimian	USA	353	yumian1	HY
304	jimian11	HY	329	611bo	Others	354	yumian2	HY
305	jimian12	HY	330	annong121	HZY	355	xinluzao53	NW
306	emian16	HZY	331	daizimian16	USA			
307	emian17	HZY	332	dezimian531	USA			
308	sumian4	HZY	333	dunmian1	NW			
309	ekangmian2	HZY	334	dunmian2	NW			
310	ekangmian3	HZY	335	ganmian2	HZY			
311	ekangmian6	HZY	336	ganmian3	HZY			
312	edaimian	HZY	337	ganmian47	HZY			
313	xuzhou142	HZY	338	Deltapine-gy	USA			
314	sumian9	HZY	339	ji668	HY			
315	sumian12	HZY	340	jimian25	HY			
316	sukang191	HZY	341	jinmian5	LN			
317	gangmian1	HZY	342	keke1543	Others			
318	gangmian2	HZY	343	ningmian1	HZY			
319	kezi201	USA	344	ningmian22	HZY			
320	daihongdai	HZY	345	nongda94-7	HY			
321	yishuhong	HZY	346	nongdamian8	HY			
322	bomian1	HY	347	shumian1	HZY			
323	kemian4	HZY	348	sumian1	HZY			
324	ganmian11	HZY	349	sumian22	HZY			
325	ganmian12	HZY	350	xiangmian10	HZY			

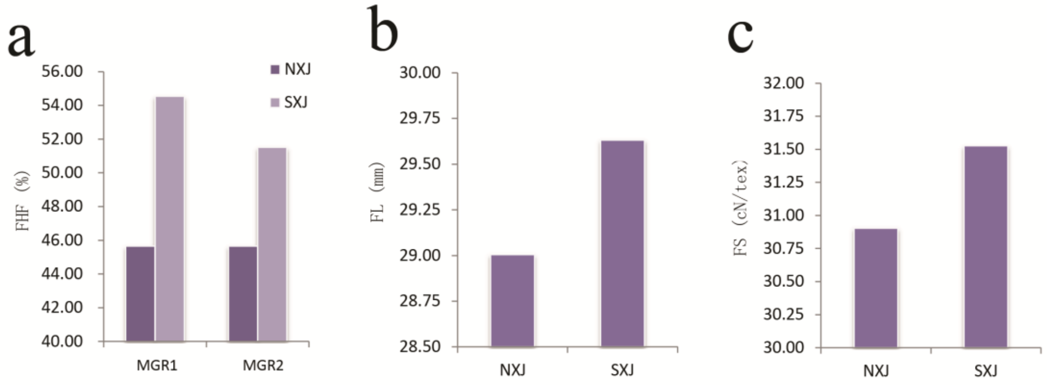

Supplementary Fig. S1. Geographic distribution and phenotypic values of the favorable haplotypes (FHs) in Xinjiang, China. (a) Favorable haplotypes frequency (FHF) of the two geographic areas in Xinjiang. (b and c) Phenotypic values of FL and FS of the two geographic areas in Xinjiang.

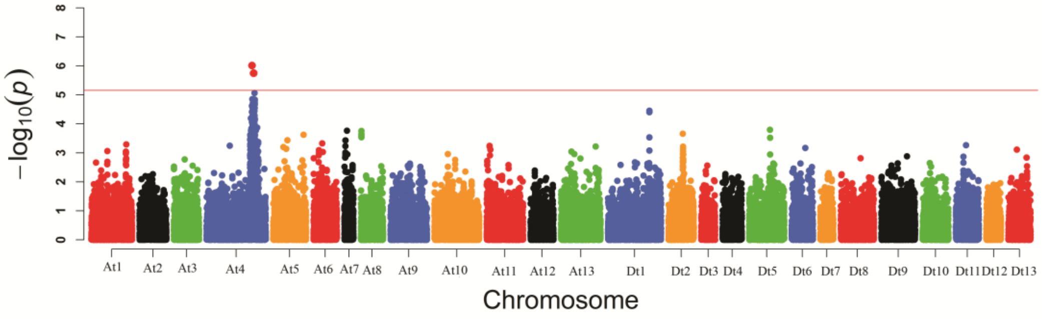

Supplementary Fig. S2. Manhattan plots of the genome-wide association studies (GWASs) of NW upland cotton accessions for fiber strength.
